# Design and mechanistic insight into ultrafast calcium indicators for monitoring intracellular calcium dynamics

**DOI:** 10.1038/srep38276

**Published:** 2016-12-06

**Authors:** Nordine Helassa, Borbala Podor, Alan Fine, Katalin Török

**Affiliations:** 1 Molecular and Clinical Sciences Research Institute, St George’s, University of London, Cranmer Terrace, London SW17 0RE, UK; 2 Department of Physiology and Biophysics, Dalhousie University, Halifax, Nova Scotia, Canada

## Abstract

Calmodulin-based genetically encoded fluorescent calcium indicators (GCaMP-s) are powerful tools of imaging calcium dynamics from cells to freely moving animals. High affinity indicators with slow kinetics however distort the temporal profile of calcium transients. Here we report the development of reduced affinity ultrafast variants of GCaMP6s and GCaMP6f. We hypothesized that GCaMP-s have a common kinetic mechanism with a rate-limiting process in the interaction of the RS20 peptide and calcium-calmodulin. Therefore we targeted specific residues in the binding interface by rational design generating improved indicators with GCaMP6f_*u*_ displaying fluorescence rise and decay times (*t*_1/2_) of 1 and 3 ms (37 °C) *in vitro*, 9 and 22-fold faster than GCaMP6f respectively. In HEK293T cells, GCaMP6f_*u*_ revealed a 4-fold faster decay of ATP-evoked intracellular calcium transients than GCaMP6f. Stimulation of hippocampal CA1 pyramidal neurons with five action potentials fired at 100 Hz resulted in a single dendritic calcium transient with a 2-fold faster rise and 7-fold faster decay time (*t*_1/2_ of 40 ms) than GCaMP6f, indicating that tracking high frequency action potentials may be limited by calcium dynamics. We propose that the design strategy used for generating GCaMP6f_*u*_ is applicable for the acceleration of the response kinetics of GCaMP-type calcium indicators.

Genetically encoded Ca^2+^ indicators (GECI) facilitated monitoring Ca^2+^ dynamics in intact and even freely moving animals. A limiting factor in the application of GECI has been their high Ca^2+^ affinity and slow response kinetics^1^. In GCaMP-type GECI^2^,^3^,^4^,^5^,^6^,^7^,^8^,^9^,^10^,^11^ Ca^2+^ binding to calmodulin (CaM) induces the formation of a complex with a target peptide which in turn restores the fluorescence of circularly permutated (cp) EGFP^12^. The Ca^2+^-induced binding of CaM to a target peptide allows the detection of Ca^2+^ signals also by GCaMP-related G-GECO-s^13^, GEM-GECO-s^13^ and sub-cellularly targeted CEPIA^14^ and GCaMPer^15^. Further broadening of the approaches for non-invasive mapping of neuronal circuits is represented by the development of red fluorescent probes for optogenetic stimulation and of photoactivatable derivatives^13^,^16^,^17^,^18^. The most commonly used target peptide is RS20, a smooth muscle myosin light chain kinase-derived peptide and, in the case of RCaMP2, a CaMKKα-derived peptide^16^. GCaMP-s reported to have the greatest brightness and Ca^2+^ induced fluorescence enhancement to date are variants of a GCaMP6 generation termed GCaMP6 slow, GCaMP6 medium and GCaMP6 fast (GCaMP6s, GCaMP6m and GCaMP6f)^19^ as well as GCaMP7^20^ and GCaMP8^21^. Even though indicators such as GCaMP7 show sufficiently high level of brightness to detect single action potentials (AP-s)^22^, their slow kinetic properties due to the Ca^2+^ - CaM - RS20 peptide interaction limit achieving temporal fidelity. Typically, Ca^2+^ - CaM - RS20 interactions have a high affinity (half-maximal brightness concentration, *K*_d_ values of 100–300 nM) as for GCaMP6s and GCaMP6f^19^. Correspondingly, the Ca^2+^ dissociation rates and thus the signal decay rates are slow, 1–5 s^−1^ at 20 °C, comparable to that of GCaMP3^10^. By rational design, mutations at the Ca^2+^ - CaM - RS20 binding interface have produced GCaMP3- and GCaMP6f-derived probes with improved decay kinetics^23^,^24^. The probe with the fastest signal rise (*t*_1/2_ of 1 ms) and decay (*t*_1/2_ of 3 ms) kinetics (37 °C) so far is GCaMP3_*fast*_^25^. The mutation strategy that gave rise to GCaMP3_*fast*_ can serve as a template for generating fast-response CaM-based GECI.

## Design template for ultrafast GCaMP-s

**Figure 1:**
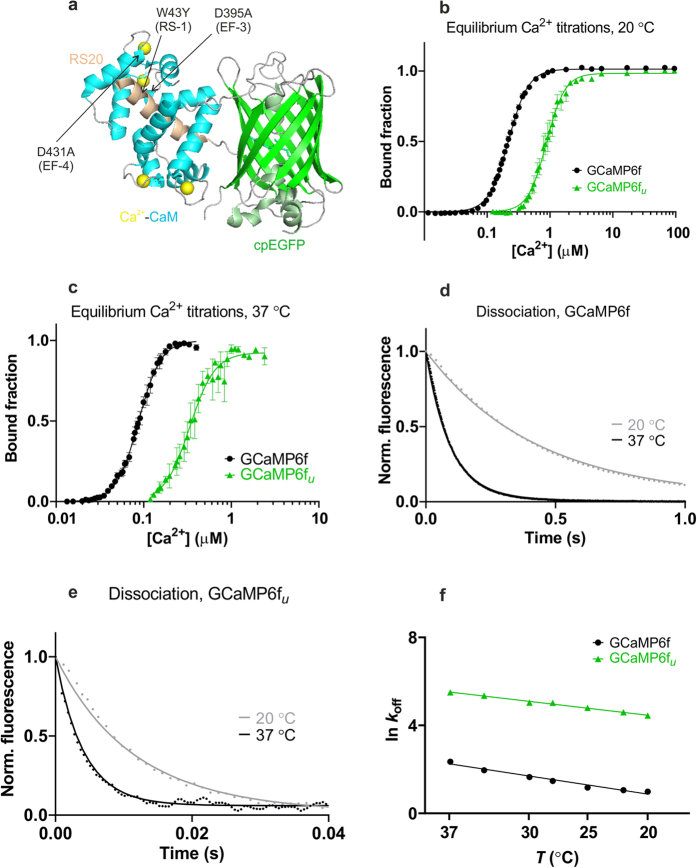
Targeted mutagenesis and biophysical characterisation of GCaMP6f and GCaMP6f_u_. (**a**) Crystal structure of monomeric GCaMP6m in a Ca^2+^-bound form with cpEGFP (green), Ca^2+^ ions (yellow), CaM (blue) and the RS20 peptide (light brown) (adapted from Ding *et al*.,^27^ PDB 3WLD). The positions of the mutated amino acid residues in the CaM EF-hand Ca^2+^-binding sites and in the RS20 peptide are highlighted. Equilibrium Ca^2+^ titrations for GCaMP6f (•) and GCaMP6f_*u*_ (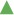), (**b**) at 20 °C (**c**) at 37 °C. Fluorescence changes are normalised to *F*_0_ of 0 and *F*_max_ of 1 and fitted to the Hill equation. Fitted curves are represented by solid lines overlaying the data points. Ca^2+^ dissociation kinetics of (**d**) GCaMP6f and (**e**) GCaMP6f_*u*_ at 20 °C (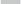) and 37 °C (**—**); Experimental data are overlaid by fitted curves using parameters for the kinetic model for GCaMPs (Supplementary Table S3). (**f**) Arrhenius plots of the observed rates for Ca^2+^ dissociation of GCaMP6f (•) and GCaMP6f_*u*_ (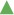).

We developed a strategy to generate fast-response GCaMP-type GECI by introducing specific mutations at the Ca^2+^ - CaM - RS20 peptide interface to weaken their interactions. In CaM, Ca^2+^ binding sites are disabled by asingle-point mutation of the first Asp to Ala in each EF-hand Ca^2+^ binding loop. The EF-3 mutation denotes a mutant with the third Ca^2+^ site inactivated, EF-4 with the fourth site. A mutation termed RS-1 which substitutes the Trp residue in position 43 for Tyr in the RS20 peptide sequence, further weakens the Ca^2+^- CaM - peptide interaction^26^ (Fig. 1a). Four trends are commonly observed when the mutational strategy is applied to GCaMP3, G-GECO and GEM-GECO. First, mutation of EF-1 and/or EF-2 causes loss of fluorescence signal^24^,^25^. Second, the EF-4 and combined RS-1 EF-4 mutations consistently retain or have increased brightness and dynamic range together with faster kinetics than the parent proteins^25^. Third, GCaMP3, G-GECO and GEM-GECO probes with the EF-3:4 double mutation alone or in combination with RS-1 have low affinity (mM *K*_d_) and fast kinetics. Although the lower brightness and dynamic range of the EF-3:4 double mutants requires improvement, kinetically they are thus promising for development of extracellular or intra-organelle Ca^2+^ probes. Finally, the combination of mutations EF-3 and RS-1 results in the fastest response kinetics and has given the to-date most kinetically improved probe mGCaMP3 RS-1 EF-3 (GCaMP3_*fast*_) which has Ca^2+^ rise and decay times of 1–3 ms^25^.

We applied the strategy used to produce GCaMP3_*fast*_ to the popular bright probes GCaMP6s and GCaMP6f. Here we test the hypothesis that the EF-3, EF-4 and RS-1 mutations and their combinations result in significantly faster kinetics when applied to CaM-based GECI. We have generated twelve mutant probes, six each for GCaMP6s and for GCaMP6f^19^. Biophysical characterisation revealed that, as predicted, the fastest responding probe was given by the RS-1 (W43Y) EF-3 (D395A) mutation combination. mGCaMP6f RS-1 EF-3, termed GCaMP6f-ultrafast (GCaMP6f_*u*_) has 1 ms rise and 3 ms decay half times *in vitro* (37 °C). The temperature dependence of the fluorescence response kinetics of the probes is analyzed to enable predictions for physiological applications and for better understanding of the molecular mechanisms of GCaMP probes. A kinetic model is proposed for the processes through which fluorescence develops in CaM-based GCaMP-s. Faster response kinetics by GCaMP6f_*u*_ compared to GCaMP6f are demonstrated in ATP-stimulated cells and in neurons stimulated by high frequency action potentials.

## Results

### Biophysical properties of GCaMP6f_*u*_ and selected mGCaMP6s and mGCaMP6f

First the effects of the mutations on the fluorescence brightness and dynamic range of the probes were determined. Quantum yield (Φ_+Ca_^2+^) measurements revealed that the EF-4 and RS-1 EF-4 mutations had little effect on the Φ_+Ca_^2+^ of GCaMP6s and GCaMP6f. Using the reported value of 0.59 at 20 °C for GCaMP6f^19^, we obtained a Φ_+Ca_^2+^ value of 0.58 for GCaMP6s and 0.46 for GCaMP6f_*u*_. With measured molar extinction coefficients *ε*_o(497 nm)_ of 50294 ± 259 M^−1^cm^−1^ for Ca^2+^-saturated GCaMP6f and 1564 ± 1140 M^−1^cm^−1^ for Ca^2+^-saturated GCaMP6f_*u*_, brightness values of 29673 and 7197 M^−1^cm^−1^ were obtained for GCaMP6f and GCaMP6f_*u*_, respectively. Similarly, Ca^2+^-induced fluorescence increases (*F*_r_(_+Ca_^2+^)), expressed relative to apo-GCaMP6s (*F*_r_(_−Ca_^2+^) of 1)) of 27 for GCaMP6s and 15 for GCaMP6f^19^, were little affected by the EF-4 and RS-1 EF-4 mutations. *F*_r(+Ca_^2+^)/*F*_r(−Ca_^2+^) of GCaMP6s RS-1 EF-3 and GCaMP6f_*u*_ were reduced to 18 and 5, respectively (Table 1 and Supplementary Table S1). Even though the brightness for GCaMP6f_*u*_ is 4-fold reduced compared to GCaMP6f, the value remains comparable to GCaMP3 probe which has been widely used for *in vivo* experiments including calcium measurements in free-moving animals (*F*_max_/*F*_min_ for GCaMP6f = 14.6, GCaMP6f_*u*_ = 5.1 and GCaMP3 = 6.3)^25^. pKa values of ~7 were measured for GCaMP6s and GCaMP6f together with their mutants at 20 °C in agreement with previously reported values (Supplementary Fig. S1)^19^.

#### Equilibrium Ca^2+^ binding

The mutations were expected and found to increase the equilibrium half-maximal brightness concentration (*K*_d_) values of the probes. Cooperativity for Ca^2+^ remained high, characterised by Hill coefficient (*n*) values of ~2–3. Comparison of affinities at 20 °C and 37 °C revealed that *K*_d_-s decreased with increasing temperature: for GCaMP6f, from 220 nM to 88 nM, respectively (Fig. 1b), for GCaMP6f_*u*_ from 890 nM to 340 nM (Fig. 1c, Table 1) and for the lower affinity mGCaMP6f EF-4, from 1.6 μM to 840 nM (Supplementary Fig. S2,S3, Supplementary Table S1).

mGCaMP6f RS-1 EF-4 and the EF-3:4 double mutants of both GCaMP6s and GCaMP6f revealed two independent sets of Ca^2+^ binding sites: high affinity (1–6 μM *K*_d_) with *n* of 2 and low affinity (0.2–6 mM *K*_d_) with *n* of 1. The high affinity *K*_d_ decreased with temperature while the low affinity *K*_d_ increased. Moreover, while 80% of the total fluorescence enhancement of mGCaMP6f RS-1 EF-4 occurred with high affinity at 20 °C, at 37 °C, a temperature closer to those in physiological applications, the lower affinity component dominated representing 70% of the signal (Supplementary Fig. S3 and Supplementary Table S1, S2). The apparent binding to three sites shown by the EF-3:4 double mutants may be explained by residual Ca^2+^ binding to a mutated C-lobe site.

#### Ca^2+^ decay kinetics

Typically, faster fluorescence decay rates were expected for the EF-3, EF-4 and RS-1 mutants of GCaMP6s and GCaMP6f compared to their parent proteins at all temperatures (Supplementary Fig. S4, S5). *t*_1/2_ at 20 °C of 769 ms for GCaMP6s was reduced to 216 ms by the RS-1 EF-3 mutation. The same mutation in GCaMP6f, giving GCaMP6f_*u*_ brought down the decay *t*_1/2_ from 288 ms to 7.8 ms (Fig. 1e, Table 1 and Supplementary Table S1).

In spite of decreasing *K*_d_ values by increased temperatures, the fluorescence decay rates were 2–5-fold increased at 37 °C compared to 20 °C (Supplementary Fig. S4,S5 and Supplementary Tables S1,S2). At 37 °C, the fastest mGCaMP6s probe, mGCaMP6s RS-1 EF-3, had a 6.5-fold increased Ca^2+^ decay rate compared to GCaMP6s with the *t*_1/2_ of 346 ms reduced to 53 ms. *t*_1/2_ at 20 °C of 288 ms of GCaMP6f was reduced to 63 ms at 37 °C (Fig. 1d). The most significant improvements in the Ca^2+^ dissociation kinetics were however achieved by the RS-1 EF-3 mutations of GCaMP6f yielding the probe GCaMP6f_*u*_ with a 23-fold faster fluorescence decay *t*_1/2_ of 3 ms at 37 °C (Fig. 1f and Supplementary Fig. S6).

#### Ca^2+^ rise kinetics

**Figure 2:**
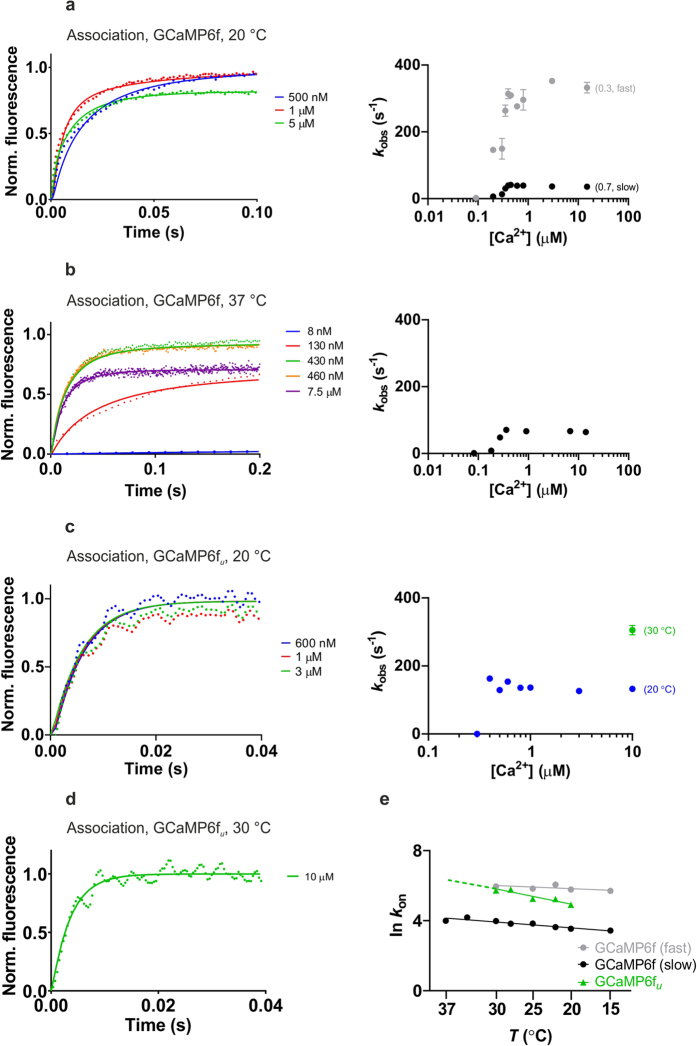
Ca^2+^ association kinetics of GCaMP6f at (**a**) 20 °C and (**b**) 37 °C; (**c**) GCaMP6f_*u*_ at 20 °C. Left hand panels show stopped-flow records at the specified final [Ca^2+^] values. Fluorescence changes are normalised to *F*_0_ of 0 and maximum of 1. Experimental data (dotted lines) are overlayed with fitted curves (solid lines) generated using the parameters shown in Supplementary Table S3 fitted to the kinetic model for GCaMP-s. Right hand panels show plots of the [Ca^2+^] dependence of the observed rate(s) (*k*_obs_). GCaMP6f has biphasic association kinetics at 20 °C; the fast phase (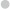) corresponds to 30% and the slow phase (•) to 70% of the fluorescence amplitude. At 37 °C, GCaMP6f Ca^2+^ association kinetics are monophasic with the fluorescence response shown only by the slow phase. (**d**) Stopped-flow record of GCaMP6f_*u*_ Ca^2+^ association at 10 μM final [Ca^2+^] measured at 30 °C, fitted to the model. (**e**) Arrhenius plots of the observed rates for Ca^2+^ association of GCaMP6f fast phase (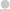), slow phase (•), GCaMP6f_*u*_ (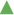). For GCaMP6f the amplitude of the fast phase diminishes with increasing temperature: relative amplitudes are at 20 °C, fast (0.3) slow (0.7); at 25 °C, fast (0.2) slow (0.8); at 30 °C, fast (0.1) slow (0.9) and at 37 °C, fast (0) slow (1.0).

Stopped-flow fluorimetry revealed biphasic Ca^2+^ association kinetics for both GCaMP6s and GCaMP6f and for most of their mutants at 20 °C. The exceptions were mGCaMP6s RS-1 EF-4, GCaMP6f_*u*_ and mGCaMP6f EF-3:4 which had a monophasic fluorescence rise (Fig. 2 and Supplementary Fig. S7, S8). [Ca^2+^] dependency of the rise rates revealed a switch-like response with limiting rates for each phase (*k*_on(lim)_). The fastest rate was measured for GCaMP6f_*u*_ which, responding in an all-or-none manner to [Ca^2+^] rise, had a single rise *t*_1/2_ of 5 ms at 20 °C (Fig. 2c).

Examination of the temperature dependence of the Ca^2+^ rise kinetics reveals the kinetic advantage of GCaMP6f_*u*_ over GCaMP6f at 37 °C (Fig. 2 and Supplementary Fig. S9). The fast phase of Ca^2+^ induced fluorescence enhancement of GCaMP6f has a limiting rate of 315 s^−1^ (rise *t*_1/2_ 2 ms) and at 20 °C represents 30% of the fluorescence enhancement. The rate of the fast phase is essentially independent of temperature in the range of 20 °C to 30 °C, however with a diminishing amplitude of only 10% at 30 °C and no longer measurable at 37 °C. The rate of the slow phase is ~2-fold increased from 38 s^−1^ to 66 s^−1^ over the temperature range of 20 °C to 37 °C, representing the only measurable rate for GCaMP6f at 37 °C. The apparent Ca^2+^ response of GCaMP6f is thus slower at 37 °C than 20 °C. In contrast, the single observed limiting rate for mGCaMP6f RS-1 EF-3 (GCaMP6f_*u*_) is ~4-fold increased, to an extrapolated 546 s^−1^ at 37 °C, from 142 s^−1^ at 20 °C giving a fluorescence rise time of 1 ms.

Complexities are revealed in the temperature dependencies of the Ca^2+^ rise kinetics of GCaMP6s and mGCaMP-s as well (Fig. 2, Supplementary Fig. S10). In the case of GCaMP6s, the relative amplitudes of the fast and slow phases change with temperature such that at physiological ionic strength and 20 °C the amplitudes are about even, however at 37 °C the slow phase (rise *t*_1/2_ 12 ms) represents 90% of the signal (Supplementary Table S1). Arrhenius plots for mGCaMP6f RS-1 EF-4 reveal opposite temperature dependencies for the rate of the fast and slow phases of its biphasic Ca^2+^ association kinetics: while the rate of the fast phase is 2-fold increased, the rate of the slow decay component is 2.5-fold lowered when the temperature increases from 20 °C to 37 °C (Supplementary Fig. S11d and Supplementary Table S1).

#### Kinetic mechanism of GCaMP-type GECI

**Figure 3:**
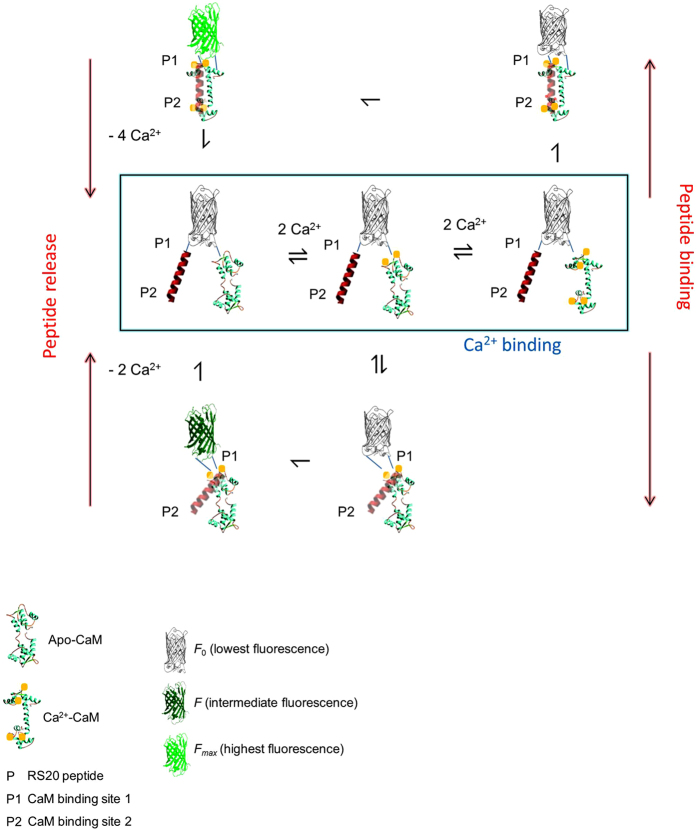
Schematic model of the kinetic mechanism of GCaMPs. General model for the mechanism of Ca^2+^-induced fluorescence of GCaMP-type probes depicting Ca^2+^ binding to the CaM N- and C-lobe followed by peptide binding and isomerisation leading to fluorescent states. Fluorescence is proposed to derive from the equilibrium of two states: in the first, Ca^2+^-bound CaM N-lobe is with the P1 CaM binding site of RS20; in the second, both the N- and C-lobes of CaM are Ca^2+^-bound binding to both the P1 and P2 sites of the peptide. Formation of the fluorescent complexes involves essentially irreversible processes. Peptide release is triggered by Ca^2+^ sequestration returning the complex to the apo-state. This general model for GCaMPs is applicable to EF-hand mutants by omitting the appropriate Ca^2+^ binding steps e.g. eq. 1 for the EF-1 mutant, eq. 2 for EF-2 and so on^25^.

As the fluorescence increase evoked by Ca^2+^ is based on the effect of the Ca^2+^ - CaM - RS20 peptide interaction on the structure of the cpEGFP domain, a common reaction mechanism previously described for GCaMP3 and its fast variants is expected to operate for GCaMP-s in general^25^. In essence, partially and fully Ca^2+^- and peptide-bound intermediates create two main fluorescent states in the rise phase of Ca^2+^-induced fluorescence. In the decay phase evoked by Ca^2+^ sequestration, Ca^2+^ leaving initiates dissociation of the complex and the return to the resting low-fluorescence state (Fig. 3). Fitted curves to the stopped-flow kinetic Ca^2+^ association and dissociation records obtained at 20 °C, 30 °C and 37 °C for GCaMP6f and GCaMP6f_*u*_ were generated using a set of fitted parameters consistent with the data (Fig. 2 and Supplementary Table S3).

### Fluorescence dynamic range measurements of GCaMP6f and GCaMP6s variants in HEK293T cells

Fluorescence dynamic range values for mGCaMP6s and mGCaMP6f probes were measured in ionomycin stimulated HEK293T cells. GCaMP6s continued to have the greatest fluorescence enhancement with Δ*F*/*F*_0_ of 18. In contrast to Δ*F*/*F*_0_ for GCaMP6f of 11, GCaMP6f_*u*_ had Δ*F*/*F*_0_ of ~ 7. Fluorescence dynamic range values obtained in cellular environment were generally reduced compared to those in solution, except for GCaMP6f_*u*_, and for mGCaMP6s EF-3:4 (Supplementary Fig. S12).

### Imaging intracellular Ca^2+^ release dynamics in ATP-stimulated HEK293T cells by GCaMP6f, GCaMP6f_*u*_ and mGCaMP6f RS-1 EF-4

**Figure 4:**
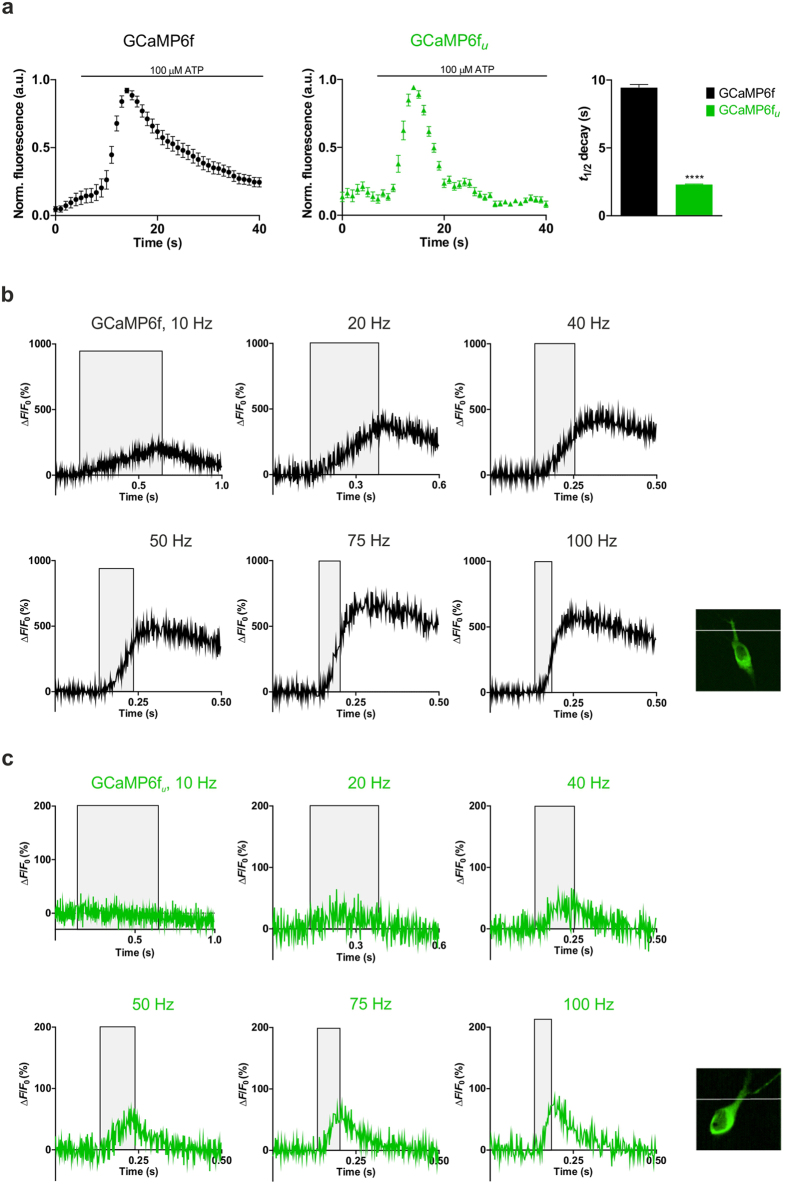
Ca^2+^ response of GCaMP6f and GCaMP6f_*u*_ in ATP-stimulated HEK293T cells and post-synaptic CA1 neurons in hippocampal slices. (**a**) Ca^2+^ transients were triggered by exposure of HEK293T cells to 100 μM ATP. Time courses of response of GCaMP6f and GCaMP6f_*u*_ are shown. *t*_1/2_ for GCaMP6f_*u*_ 2.3 ± 0.04 s was significantly different from that for GCaMP6f (9.4 ± 0.2 s). (**b**) Ca^2+^ response kinetics in post-synaptic CA1 neurons in hippocampal slices of GCaMP6f (28 °C) to stimulation by 5 action potentials (AP-s). Grey shaded areas indicate the duration of stimulation. GCaMP6f stimulated at (3 cells, n number of recordings in brackets) 10 Hz (n = 28), 20 Hz (n = 28), 40 Hz (n = 29), 50 Hz (n = 27), 75 Hz (n = 30) and 100 Hz (n = 19); (**c**) GCaMP6f_*u*_ stimulated (4 cells, n number of recordings in brackets) at 10 Hz (n = 29), 20 Hz (n = 30), 40 Hz (n = 29), 50 Hz (n = 29), 75 Hz (n = 30) and 100 Hz (n = 26). The achieved maximum Δ*F*/*F*_0_ values are plotted against time. Inset images: representative images baseline expression of GCaMP6f and GCaMP6f_*u*_ in CA1 pyramidal neurons with white line in the position of the line scan.

Intracellular Ca^2+^ dynamics were monitored in ATP-stimulated HEK293T cells and the time courses of the Ca^2+^ transient evoked by GCaMP6f, GCaMP6f_*u*_ and mGCaMP6f RS-1 EF-4 were compared. GCaMP6f fluorescence increased (Δ*F*/*F*_0_ of 3.1 ± 0.35) with a decay time *t*_1/2_ of 9.4 ± 0.2 s. In comparison, GCaMP6f_*u*_ and mGCaMP6f RS-1 EF-4 both showed lower dynamic ranges (Δ*F*/*F*_0_ of 0.2 ± 0.02) and significantly reduced, 2.3 ± 0.04 s decay time. Faster probes GCaMP6f_*u*_ and mGCaMP6f RS-1 EF-4 thus revealed that ATP-induced intracellular Ca^2+^ release dynamics were more rapid than reported by GCaMP6f (Fig. 4a).

### Response of GCaMP6f, GCaMP6f_u_ and mGCaMP6f RS-1 EF-4 to high frequency stimulation in neurons

GCaMP6f, GCaMP6f_*u*_ and mGCaMP6f RS-1 EF-4 expressed in hippocampal CA1 pyramidal neurons in organotypic hippocampal slices were distributed homogenously. Baseline fluorescence of all three indicators was bright enough to readily identify expressing cells. Fluorescence dynamic ranges (Δ*F*/*F*_0_) were 5.9 ± 0.3, 0.8 ± 0.05 and 0.5 ± 0.04 for GCaMP6f, GCaMP6f_*u*_ and mGCaMP6f RS-1 EF-4, respectively, consistent with small [Ca^2+^] changes. Notably, GCaMP6f achieved a significantly higher Δ*F*/*F*_0_ value throughout the 10–100 Hz range tested compared to GCaMP6f_*u*_ and mGCaMP6f RS-1 EF-4. Due to its low *K*_d_ for Ca^2+^, only GCaMP6f was able to detect the low post-synaptic [Ca^2+^] elevation evoked by 5 AP-s fired at 10 Hz. For the same reason, however, the kinetics of GCaMP6f response were the most prolonged. With their higher K_*d*_ values GCaMP6f_*u*_ and mGCaMP6f RS-1 EF-4 did not show a response to stimulation up to 20–40 Hz frequency and the dynamic range remained low. The kinetic advantage of GCaMP6f_*u*_ and mGCaMP6f RS-1 EF-4 became evident at stimulation at 40 Hz or greater (Fig. 4b,c and Supplementary Fig. S13) and was particularly clear at 100 Hz: compared to GCaMP6f, GCaMP6f_*u*_ and mGCaMP6f RS-1 EF-4 responded with much faster rise and decay kinetics to the [Ca^2+^] elevation evoked by trains of 5 AP-s. For GCaMP6f, following 50 ms stimulation at 100 Hz, the Ca^2+^ signal is still rising for another ~ 75 ms and then decays over ~ 400 ms^19^ (Fig. 4b). mGCaMP6f RS-1 EF-4 has improved kinetics with the signal decaying only~20 ms after stimulation, with a *t*_1/2_ of 91 ± 17 ms (Supplementary Fig. S13). The fastest response is achieved by GCaMP6f_*u*_: the Ca^2+^ signal decay commences as soon as the stimulation is finished, and returns to baseline with a *t*_1/2_ of 40 ± 2 ms, thus reducing the refractory period of detection following a stimulation event from hundreds to tens of milliseconds (Fig. 4c).

## Discussion

The aim of this work was to reveal generally applicable modifications for the generation of fast-response GCaMP-like GECI for more accurate time resolution of Ca^2+^ signalling events. The combination of RS-1 and EF-3 mutations gave mGCaMP3 RS-1 EF-3 (GCaMP3_*fast*_) which has a 1 ms rise and 3 ms decay *t*_1/2_ at 37 °C^25^. The application of GCaMP3_*fast*_ in cardiac myocytes revealed faster Ca^2+^ transients then previously indicated by slow indicators^25^. Here we report that the same design template when used on GCaMP6f results in similarly fast on and off response kinetics and propose that the RS-1 EF-3 mutation combination is applicable for the generation of ultrafast GCaMP-s.

**Figure 5:**
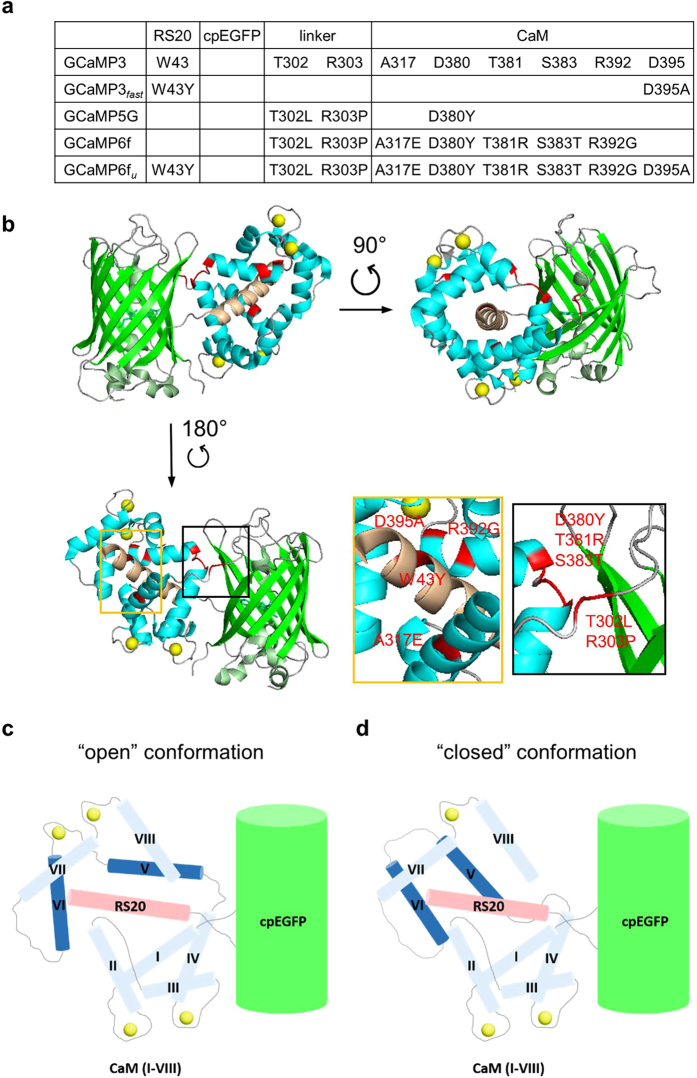
Comparison of fast GCaMP probes (GCaMP3_*fast*_ and GCaMP6f_*u*_) and their parent variants (GCaMP3 and GCaMP6f). (**a**) Position of the mutations that gave rise to GCaMP3_*fast*_ and GCaMP6f_*u*_ variants, relative to GCaMP3. (**b**) Crystal structure of monomeric GCaMP6m in a Ca^2+^-bound form with cpEGFP (green), Ca^2+^ ions (yellow), CaM (blue) and the RS20 peptide (light brown) (adapted from Ding *et al*.,^27^ PDB 3WLD). The amino acid residues highlighted in red are those that generated GCaMP6f_*u*_ relative to GCaMP3. Schematic representation of (**c**) Ca^2+^-bound GCaMP3/GCaMP6f showing “open” conformation of EF-hand 3 (helices V and VI); (**d**) Ca^2+^-bound GCaMP3_*fast*_/GCaMP6f_*u*_ showing “closed” conformation of disabled EF-hand 3 (helices V and VI).

While the RS-1 (W43Y) EF-3 (D395A) mutation pair has equal effects on the kinetics of GCaMP3 and GCaMP6f, they differently affect the brightness and dynamic range of their parent variants: GCaMP3_*fast*_ retains the brightness of GCaMP3, whereas GCaMP6f_*u*_ is 4-fold decreased compared to GCaMP6f. Even though GCaMP6m is significantly brighter than GCaMP3, the alignment of their crystal structures (PDB codes 3SG3 and 3WLD) reveals only subtle differences in the position of helix I and the linker region to cpEGFP^3^,^27^. The brightness and fluorescence dynamic range of GCaMP6f are higher than of GCaMP3 because of the introduction of the ‘stabilising’ mutations in the linker region connecting the CaM N-terminus to cpEGFP (T302L, R303P) and in helices I, IV and V of CaM (A317E, D380Y/T381R/S383T, R392G, respectively) (Fig. 5a,b). In particular, the T302L, R303P, D380Y hydrophobic mutations at the interface between CaM and cpEGFP result in similarly increased brightness and dynamic range in GCaMP5G and GCaMP6f^3^,^19^. The gain of brightness is likely due to a structural rearrangement involving these crucial hydrophobic residues not present in GCaMP3 as described below. These mutations decrease the flexibility of the cpEGFP - CaM helix I linker, through interaction between helix IV-V and cpEGFP - CaM helix I linkers. Specifically, the T302L, R303P, D380Y mutations play the most significant roles in ordering/positioning cpEGFP - CaM helix I linker, as GCaMP5G is almost as bright as GCaMP6f. The R303P mutation decreases flexibility and increases order in the cpEGFP - CaM helix I linker, thus positioning the mutated L302 and Y380 hydrophobic sidechains in proximity allowing them to form tertiary interactions. In GCaMP6f, the cpEGFP - CaM helix I and helix IV-V linkers interact and stabilise one another. Thus the three mutations position the cpEGFP - CaM helix I linker with respect to RS-20 peptide - cpEGFP linker stabilising cpEGFP folding. In contrast, the T302, R303 and D380 residues, originally present in GCaMP3, allow the cpEGFP - CaM helix I linker to be more dynamic, thus increasing entropic penalty for cpEGFP folding in GCaMP3 with respect to GCaMP6f. In GCaMP3, the cpEGFP - CaM helix I linker and helix IV-V linkers are dynamic, reducing the stability of folded cpEGFP, explaining/causing its reduced brightness, which is the measure of the fraction of folded cpEGFP. The D395A mutation, introduced in GCaMP3_*fast*_ and GCaMP6f_*u*_, prevents Ca^2+^ binding to the EF-3 site (flanked by helices V and VI), which thus, rather than being in an “open” conformation as in Ca^2+^-bound GCaMP-s (Fig. 5c), is expected to remain in “closed” conformation (Fig. 5d). Thus, the cpEGFP - CaM helix I linker is imperfectly poised with respect to RS-20 peptide - cpEGFP linker, cpEGFP folding is thus destabilised and the brightness is reduced in GCaMP3_*fast*_ and GCaMP6f_*u*_. In GCaMP3, the CaM EF-hand 3 adopts “open” conformation, but the cpEGFP - CaM helix I linker remains dynamic, hence cpEGFP folding is still destabilised explaining its similar brightness to GCaMP3_*fast*_ and GCaMP6f_*u*_. Closing the CaM EF-hand 3 conformation by the W43Y/D395A mutation thus has the equivalent effect on cpEGFP folding as destabilising the cpEGFP - CaM helix I linker.

We observed that GCaMP-type GECI have a limiting response rate that is independent of [Ca^2+^]^25^. Moreover, the Ca^2+^ rise kinetics of GCaMP6s and GCaMP6f revealed complex biphasic processes: although at 20 °C GCaMP6s and GCaMP6f have 20 ms and 2 ms response times, the amplitude of their fast phase diminishes with increasing temperature, thus presenting as slow indicators, at 37 °C. In contrast, GCaMP3_*fast*_ and GCaMP6f_*u*_ have greatly improved fluorescence rise time of 1 ms *t*_1/2_ at 37 °C. By targeting the CaM - RS20 peptide interface, GCaMP3 RS09 and GCaMP6f RS09 had improved decay *t*_1/2_-s of 25 and 20 ms (37 °C)^23^,^24^, compared to decay *t*_1/2_-s of GCaMP3 with 147 ms and GCaMP6f with 71 ms. However GCaMP3_*fast*_^25^ and GCaMP6f_*u*_ have the fastest decay with a *t*_1/2_ of 3 ms, at 37 °C.

The fluorescence response kinetics of GCaMP-s have been modelled from the stopped-flow data. Previously a model has been put forward in which the Ca^2+^ response rate depends on the Ca^2+^ step: a small Ca^2+^ step results in slow binding to the C-lobe followed by fast binding to the low affinity the N-lobe; in contrast, a large step in Ca^2+^ evokes fast Ca^2+^ response via N-lobe binding and in this mode C-lobe binding is not required for the fluorescent state^24^. Our modeling studies reveal a different mechanism: first, that although N-lobe Ca^2+^ binding is necessary for Ca^2+^-induced fluorescence enhancement, it is not sufficient for the fluorescence response which also requires C-lobe Ca^2+^-binding; second, the limiting rate of the probes is determined by the Ca^2+^ association kinetics of the C-terminal CaM lobe in conjunction with the re-equilibration of the two postulated fluorescent states. Ca^2+^ binding is of lower affinity to the CaM N-lobe than to the C-lobe, however, in the presence of a target peptide the Ca^2+^ affinity of the N-lobe may be similar or higher than that of the C-lobe^28^. The Ca^2+^ binding kinetics of CaM-peptide complexes have not been determined, thus the rate constants for CaM were used^29^. Ca^2+^ dissociation from CaM and its peptide complexes is commonly thought to occur in pairs of Ca^2+^. Whether a single or double exponential process is observed for the fluorescence decay depends on the rates by which the putative two- and four-Ca^2+^ saturated states convert into each other and decay^26^.

Temperature dependencies of the Ca^2+^ rise kinetics suggest complex underlying activation thermodynamics. Positive, neutral and negative temperature-induced rate changes all occur, indicating that the EF-hand and peptide mutations have sensitively affected the balance of entropy and enthalpy gains. Favourable dehydration of hydrophobic surfaces results in entropy-driven activation and a positive temperature coefficient^30^. The lack of temperature dependence of the rates, e.g. GCaMP6f fast on-rate, is consistent with the limiting fast rate being Ca^2+^ binding, a diffusion limited process. Negative temperature coefficients observed for EF-3 and EF-3:4 mutants likely stem from less hydrophobic interaction and hence less positive entropy and more negative enthalpy change deriving from CaM C-lobe interaction with peptide in the absence of EF-3 or EF-4 Ca^2+^ binding and weakened binding of peptide due to W to Y substitution. Reduced brightness appears to be a trade-off for fast kinetics, presenting an opportunity for future improvements. While the EF-3 Ca^2+^ binding site is responsible for brightness, the EF-4 site is the guardian of cooperativity of the C-terminal CaM lobe for Ca^2+^ when binding the RS20 target peptide.

A commonly observed property of cpEGFP- and CaM-peptide interaction-based GECI is high cooperativity for Ca^2+^, often showing an all-or-none response to Ca^2+^ elevation as revealed by stopped-flow kinetic experiments on GCaMP3_*fast*_^25^ and GCaMP6f_*u*_. Fast all-or-none response to small changes in [Ca^2+^] would make GCaMP-type probes well suited for spike detection, however the current fast variants have insufficient brightness. RCaMP2 and some mGCaMP3, mG-GECO and mGEM-GECO have slower kinetics and reduced cooperativity for Ca^2+^ with Hill coefficients of 1–1.7 16,25, possibly by a combination of negatively and positively cooperative molecular responses, making them potentially useful for quantifying [Ca^2+^] changes in cells.

While the EF-3 single mutation raises the *K*_d_ to low μM, the EF-4 single and the EF-3:4 double mutations break up the affinity into two components, one with a *K*_d_ of low μM and one in the mM range with most of the fluorescence enhancement occurring in mM Ca^2+^. In the kinetic modeling only high affinity sites with μM *K*_d_ are considered. As there are only two Ca^2+^ binding sites in the EF-3:4 mutants, the low, mM affinity Ca^2+^ binding is likely attributable to the mutated EF-hand 3 (necessary for high brightness and dynamic range) which in the presence of the RS20 target peptide may acquire such affinity for Ca^2+^. EF-3:4 double EF-hand mutant probes with mM *K*_d_-s may be for suitable for intra-organelle and extracellular applications, after further improvements to their brightness and dynamic range. mGCaMP6s EF-3:4 is the most promising low affinity probe with a 500 μM *K*_d_ and a 19-fold Ca^2+^-induced fluorescence enhancement (20 °C), comparing favourably with CEPIA^14^.

The success of monitoring intracellular Ca^2+^ dynamics requires that the detection range of the probe is appropriate for the size and time course of the [Ca^2+^] transient. In the conditions of ionomycin stimulation, the expected peak [Ca^2+^] was 0.4 to 0.8 μM^31^. GCaMP6s with *K*_d_ of 110 nM, 5–10-fold lower than the peak of the [Ca^2+^] transient, was thus expected to be mostly saturated and thus signal such a transient with close to its maximum dynamic range. However, at resting [Ca^2+^] of ~ 50–80 nM, there is already considerable Ca^2+^ binding to such a probe. As a result of the two parameters the measured Δ*F*/*F*_0_ of 18 for GCaMP6s in ionomycin stimulated HEK293T cells is a reasonable value.

Regarding the kinetics of Ca^2+^ indicators, it is evident that low *K*_d_ probes with slow response report slower intracellular Ca^2+^ dynamics than higher *K*_d_ probes with fast responses. GCaMP6f_*u*_ and mGCaMP6f RS-1 EF-4 both showed 4-fold faster Ca^2+^ decay in response to ATP stimulation than GCaMP6f. By its two-fold faster response rate, in cardiac myocytes, GCaMP3_*fast*_ revealed removal of Ca^2+^ and return to resting intracellular levels between each beat, a phenomenon not clearly resolved by GCaMP6f^25^.

It transpires that the brightness and dynamic range of GCaMP6f_*u*_ are not sufficient for the detection of the post-synaptic dendritic [Ca^2+^] elevation evoked by a single AP. Although the kinetic properties of GCaMP6f_*u*_ would allow the separation of individual AP-s even at 100 Hz, the actual Ca^2+^ transient reveals an integrated Ca^2+^ response, raising the possibility that the Ca^2+^ dynamics associated with AP firing limit the use of Ca^2+^ as an indicator of neuronal activity.

Although unable to detect single AP-s, GCaMP6f_*u*_ with its fast rise and decay kinetics may still be useful for the detection of repeated AP-s with between-train intervals of tens rather than hundreds of milliseconds. The Ca^2+^ induced fluorescence rise times of GCaMP3 RS09 and GCaMP6f RS09 remained slow in physiological experiments^23^,^24^. In contrast, the Ca^2+^ rise rates following a train of 5 AP-s in organotypic hippocampal slices reported by the GCaMP6f_*u*_ and mGCaMP6f RS-1 EF-4 probes were markedly faster than GCaMP6f. When comparing the novel variant GCaMP6f_*u*_ to GCaMP3_*fast*_^25^, though they show similar kinetic properties, GCaMP6f_*u*_ which has a 3-fold lower *K*_d_ (0.89 μM) compared to GCaMP3_*fast*_ (2.8 μM) is more sensitive to small Ca^2+^ concentration changes and hence more suitable for Ca^2+^ imaging in neurons. Improved brightness and fluorescence dynamic range would help make GCaMP6f_*u*_ with its ultrafast kinetics a useful physiological tool.

## Materials and Methods

### Materials

GCaMP6s and GCaMP6f plasmids were a gift from Douglas Kim (Addgene plasmid #40753 and #40755, respectively)^19^. pET41a and pEGFP-N1 vectors were obtained from Novagen and Clontech, respectively. XL10-Gold and BL21 (DE3) Gold cells were purchased from Invitrogen. Restriction enzymes were obtained from New England Biolabs and T4 DNA ligase from Fermentas.

### Generation of improved GCaMP6 probes

The strategy previously developed and described for the improvement of GCaMP3 probes was followed to design, generate and purify mutants of GCaMP6s and GCaMP6f probes^19^. For protein expression, GCaMP6s and GCaMP6f were subcloned from pGP-CMV backbone into pET41a bacterial expression vector. Mutant GECI were generated by site-directed mutagenesis of CaM EF-hands and RS20 peptide as previously described^25^. GCaMP6s and GCaMP6f and their mutants were overexpressed as GST-fusion proteins overnight at 20 °C in the presence of 0.5 mM IPTG in *E. Coli* BL21(DE3) Gold cells. Cells were lysed by sonication and clarified lysates were purified by a single-step GST-chromatography (GSTrap, ÄKTA Purifier, GE Healthcare) in 50 mM Na^+^-HEPES pH 7.5, 200 mM NaCl and 10 mM glutathione, at 4 °C. Purity of the eluted fractions was determined by SDS-PAGE (6.4%–20% acrylamide/bisacrylamide gradient). Protein concentrations were measured spectroscopically at 280 nm by a Nanodrop 1000 spectrophotometer (Thermo Scientific) using an *ε*_o_ value of 76585 M^−1^cm^−1^ calculated from the amino acid composition for GCaMP6s, GCaMP6f and their EF-hand mutants and *ε*_o_ of 70710 M^−1^cm^−1^ for the RS-1 mutants^32^.

### Quantum yield determination

Protein concentrations were adjusted such that the absorbance at the excitation wavelength (490 nm) was between 0.001 and 0.1. A series of dilutions was prepared in a buffered solution (50 mM HEPES-K^+^ pH 7.5, 100 mM KCl, 2 mM MgCl_2_ with either 5 mM EGTA or 1 mM CaCl_2_) and the fluorescence spectra were recorded on a Fluorolog3 spectrofluorimeter (Horiba Scientific) at 20 °C. The quantum yield measured for GCaMP6f in Ca^2+^-saturated buffer was used as a reference (Φ = 0.59)^19^. Data were plotted as integrated fluorescence intensity as a function of absorbance and fitted by linear regression. The gradient of the plots for the reference GCaMP6f and the protein to be measured are termed S_GCaMP6f_ and S_protein_, respectively. Quantum yields were obtained using the following equation: Φ_protein_ = Φ_GCaMP6f_ × (S_protein_/S_GCaMP6s_).

### pH sensitivity of GCaMP6s and GCaMP6f-derived proteins

To determine the apparent p*K*a for each protein in the presence and absence of Ca^2+^, a series of buffers with 0.5 pH unit intervals were prepared. Depending on their respective pH buffering range, appropriate buffer was used for the measurements (MES for pH 6–6.5, HEPES for pH 7–8, TRIS for pH 8.5–9 and CAPS for pH 10). The pH titrations were performed by recording fluorescence spectra of 1 μM protein in Ca^2+^-free (50 mM buffer, 100 mM KCl, 2 mM MgCl_2_, 2 mM BAPTA) or Ca^2+^-saturated (50 mM buffer, 100 mM KCl, 2 mM MgCl_2_,1 mM CaCl_2_) conditions (Fluorolog3, Horiba). BAPTA was chosen over EGTA as Ca^2+^ chelator because of its stable affinity for Ca^2+^ over the pH range^33^. All titrations were performed in triplicates and expressed as normalised mean ± s.e.m.

### Determination of free Ca^2+^ concentrations ([Ca^2+^])

[Ca^2+^] were calculated using the Ca/Mg/ATP/EGTA Calculator v1 constants from Schoenmakers’ Chelator program for experiments at 20 °C and 37 °C^34^.

### Equilibrium Ca^2+^ binding

Ca^2+^ affinity assays of GECI and mGECI were performed by continuous titration using an automated syringe pump (ALADDIN 1000, WPI) at 20 ^o^C or 37 °C. GECI proteins at 50–100 nM concentration (50 mM K^+^-HEPES pH 7.5, 100 mM KCl, 2 mM MgCl_2_ and 5 mM EGTA) were titrated with 325 mM CaCl_2_ at a 10 μL/min flow rate in a stirred 3 mL cuvette. Fluorescence was measured at 492 nm excitation and 515 nm emission wavelengths on a Fluorolog3 spectrofluorimeter (Horiba Scientific). Fluorescence records were corrected for dilution and photobleaching. Data were normalised and expressed as bound fraction. Half-maximal brightness concentration (*K*_d_) and cooperativity for Ca^2+^ (*n*) were obtained by fitting the data to the Hill equation using GraphPad Prism 6 software. All titrations were performed at least in triplicates and expressed as mean ±  s.e.m.

### Stopped-flow fluorimetry

Ca^2+^ association and dissociation kinetic experiments of GCaMP6s and GCaMP6f proteins were carried out on a Hi-Tech Scientific SF-61DX2 stopped-flow system at temperatures ranging from 15 °C to 37 °C. Fluorescence excitation was set to 492 nm and fluorescence emission was collected using a 530 nm cut-off filter. Experiments were performed at least in triplicates and three sets of data were averaged for analysis. Data were fitted to either a single or a double exponential to obtain the rise or decay rates using KinetAssyst software.

#### Association kinetics

The solution containing 1 μM protein in 50 mM K^+^-HEPES pH 7.5, 100 mM KCl, 2 mM MgCl_2_ and 10 mM EGTA was rapidly mixed (1:1) with 50 mM K^+^-HEPES pH 7.5, 100 mM KCl, 2 mM MgCl_2_ and 10 mM EGTA containing increasing [Ca^2+^] concentrations (concentrations in the mixing chamber). For the determination of temperature dependence of Ca^2+^ association rates, protein samples at 1 μM concentration were mixed as above with saturating Ca^2+^ (7.5–20 μM final [Ca^2+^] in the mixing chamber).

#### Dissociation kinetics

The solution containing 1 μM protein in 50 mM K^+^-HEPES, 100 mM KCl, 2 mM MgCl_2_, pH 7.5 with saturating Ca^2+^ (0.5 mM) was rapidly mixed (1:1) with 50 mM K^+^-HEPES, 100 mM KCl, 2 mM MgCl_2_, 10 mM EGTA pH 7.5 (concentrations in the mixing chamber).

### Cloning of mGECI into a mammalian expression vector

For expression in eukaryotic cells, the GCaMP6s and GCaMP6f mutant DNAs were subcloned from pET41a into pEGFP-N1 vectors by restriction-ligation using BglII and NotI restriction enzymes and T4 DNA ligase following manufacturer’s protocol. During this process, the *egfp* initially present in the pEGFP-N1 vector was replaced by the *gcamp6* genes (without the GST tag).

### Imaging of GCaMP6f variants in HEK293T cells

Cells were cultured in DMEM containing 10% fetal bovine serum and 1% penicillin/streptomycin at 37 °C and 5% CO_2_.

#### Ionomycin stimulation

Transfection of cells was carried out on 10 mm diameter coverslips with 0.6 μg DNA and 2.5 μL Lipofectamine 2000 in 100 μL OptiMem® for 1 h at 37 °C. Cells were imaged 16–24 h post-transfection in 10 mM HEPES pH 7.5, 145 mM NaCl, 2.5 mM KCl, 1 mM MgCl_2_, 10 mM glucose and 2 mM CaCl_2_. For determination of the fluorescence dynamic range, cells were imaged before and after stimulation by 10 μM ionomycin at 20 °C. Imaging was performed with a Leica SP1 confocal microscope using a HCX ApoL 40 × 0.8 NA water immersion objective, images were acquired at 512 × 512 pixel size. Experiments were performed at least in triplicates, elliptical regions of interest (ROI) were selected and data were analysed using Leica Application Suite.

#### ATP stimulation

HEK293T cells were transfected with GCaMP6f variants using Fugene HD following the manufacturer’s recommendations. Imaging was performed 16–24 h post-transfection at 37 °C (OKO lab incubation chamber) in a 35-mm glass bottom dish (MatTek) with a 3i Marianas spinning-disk confocal microscope equipped with a Zeiss AxioObserver Z1, a 40 × 1.3 NA oil immersion objective and a 3i Laserstack as excitation light source (488 nm). Emitted light was collected through a single bandpass filter (Yokogawa CSU-X filter wheel) onto a CMOS camera (Hamamatsu, ORCA Flash 4.0; 1152 × 1656 pixels). Cells were stimulated with 100 μM ATP and images were collected at 1 s intervals for 40 seconds. Elliptical ROI were stacked using ImageJ program. Data obtained from 14–24 cells was plotted and analysed on GraphPad Prism 6. Fluorescence decay times were determined from single exponential fits to the data.

### Two-photon imaging of GCaMP6f, GCaMP6f_*u*_ and mGCaMP6 RS-1 EF-4 in hippocampal slices

Sprague Dawley rats were purchased from Charles River. All experiments were conducted in accordance with the Canadian Council on Animal Care standards and guidelines, with approval of the Dalhousie University Committee on Laboratory Animals. Transverse organotypic hippocampal slices (350  μm) were made from brains of rat pups aged 6 to 8 postnatal days humanely killed by Schedule 1 procedure (anesthetic (ketamine) overdose followed by decapitation) and cultured on Millicell CM membranes for 5 to 6 days^35^,^36^. Hippocampal slices were transfected using biolistics as described previously^37^. Slice preparations were transferred to a recording chamber and superfused at 28 °C with artificial cerebrospinal fluid (containing 120 mM NaCl, 3 mM KCl, 2 mM MgSO_4,_ 4 mM CaCl_2_, 1.2 mM NaH_2_PO_4_ and 11 mM glucose) saturated with a gas mixture of 95% O_2_/5% CO_2_.

An upright epifluorescence microscope (Olympus BX51WI) with a 60 × 1.0 NA IR water immersion objective (Olympus) and an MRC1024MP (Bio-Rad Microscience, Hemel Hampstead, United Kingdom) laser scanner with nondescanned photomultiplier tube detectors were used to view slices. Two-photon imaging was carried out 1–4 days post-transfection using a MaiTai titanium sapphire laser) at 930 nm.

Action potentials were evoked and recorded in expressing CA1 pyramidal neurons using a sharp intracellular electrode (60–80 MΩ) filled with 3 M KCl in 20 mM 2-[4-(2-hydroxyethyl)piperazin-1-yl]ethanesulfonic acid. Line scans taken at 2 ms interval across proximal apical dendrites were collected to determine the calcium transients measured as Δ*F*/*F*_0_ (defined as Δ*F*/*F*_0_ = 100 (*F*_transient_-*F*_initial_)/(*F*_initial_-*F*_background_) corresponding to 5 AP-s evoked by brief depolarizing current injection at frequencies between 10–100 Hz *via* a Multiclamp 700B amplifier (Molecular Devices, California). Ca^2+^ decay kinetics of GCaMP6 variants were determined by monitoring fluorescence changes evoked by 5 APs at 100 Hz.

Electrophysiological and imaging data were collected and analysed with software from AxoGraph (AxoGraph Scientific, Sydney, Australia), LaserSharp (Bio-Rad Microscience) and ImageJ^38^.

## Additional Information

**How to cite this article**: Helassa, N. *et al*. Design and mechanistic insight into ultrafast calcium indicators for monitoring intracellular calcium dynamics. *Sci. Rep.* **6**, 38276; doi: 10.1038/srep38276 (2016).

**Publisher's note:** Springer Nature remains neutral with regard to jurisdictional claims in published maps and institutional affiliations.

## Supplementary Material

Supplementary Materials

## Figures and Tables

**Table 1:** Comparison of the biophysical characteristics of GCaMP6f and GCaMP6f_*u*_.

		*F*_r_		*F*_r_(+Ca^2+^)/*F*_r_(−Ca^2+^)	*K*_d_ (μM)	*n*	*k*_on_ (s^−1^)	*t*_1/2(on)_ (ms)	*k*_off_ (s^−1^)	*t*_1/2(off)_ (ms)
		−Ca^2+^	+Ca^2+^							
**GCaMP6f**	20 °C	0.9 ± 0.1	14.4 ± 3.4	14.6 ± 2.4	0.22 ± 0.01	2.8 ± 0.1	315 ± 9 (0.3)^a^	2.2 ± 0.1 (0.3)^a^	2.4 ± 0.1	288 ± 12
							38 ± 1 (0.7)^a^	18 ± 0.5 (0.7)^a^		
	37 °C	1.0 ± 0.1	13.8 ± 0.1	13.2 ± 0.1	0.09 ± 0.01	3.4 ± 0.1	66 ± 1	10 ± 0.2	11 ± 1	63 ± 6
**GCaMP6f**_***u***_(mGCaMP6f RS-1 EF-3)	20 °C	1.0 ± 0.1	5.1 ± 0.4	5.1 ± 0.1	0.89 ± 0.01	3.0 ± 0.1	142 ± 4	4.9 ± 0.1	89 ± 1	7.8 ± 0.1
	37 °C	0.9 ± 0.1	3.8 ± 0.2	4.1 ± 0.2	0.34 ± 0.01	3.0 ± 0.3	546 ± 44^b^	1.3 ± 0.02^b^	245 ± 10	2.8 ± 0.1

^a^Biphasic association kinetic records were fitted with two exponentials. The rise *t*_1/2_ of each phase is given with the relative amplitudes in parentheses.

^b^Measurements were made in the range of 15 to 30 °C. The rate was too fast to measure at 37 °C and was extrapolated from the Arrhenius plot assuming the gradient remaining unchanged (Fig. 2e).
