# Corrigendum: Design and mechanistic insight into ultrafast calcium indicators for monitoring intracellular calcium dynamics

**DOI:** 10.1038/srep40971

**Published:** 2017-01-25

**Authors:** Nordine Helassa, Borbala Podor, Alan Fine, Katalin Török

Scientific Reports 6: Article number: 3827610.1038/srep38276; published online: 12 06 2016; updated: 01 25 2017

This Article contains a typographical error. In the Results section,

‘With measured molar extinction coefficients *ε*_o(497 nm)_ of 50294 ± 259 M^−1^ cm^−1^ for Ca^2+^-saturated GCaMP6f and 1564 ± 1140 M^−1^ cm^−1^ for Ca^2+^-saturated GCaMP6f_*u*_, brightness values of 29673 and 7197 M^−1^ cm^−1^ were obtained for GCaMP6f and GCaMP6f_*u*_, respectively.’

should read:

‘With measured molar extinction coefficients *ε*_o(497 nm)_ of 50294 ± 259 M^−1^ cm^−1^ for Ca^2+^-saturated GCaMP6f and 15646 ± 1140 M^−1^ cm^−1^ for Ca^2+^-saturated GCaMP6f_*u*_, brightness values of 29673 and 7197 M^−1^ cm^−1^ were obtained for GCaMP6f and GCaMP6f_*u*_, respectively’.

