# Supplementary material for: Design and mechanistic insight into ultrafast calcium indicators for monitoring intracellular calcium dynamics
Source: Sci Rep. 2016 Dec 6;6:38276. doi: 10.1038/srep38276 (PMC5138832; doi:10.1038/srep38276)
Supplement: Supplementary Materials [file srep38276-s1.pdf]

Design and mechanistic insight into ultrafast calcium indicators for monitoring intracellular calcium dynamics

Nordine Helassa[a], Borbala Podor[b], Alan Fine[b] and Katalin Török[a]*

[a]Molecular and Clinical Sciences Research Institute, St George's, University of London, Cranmer Terrace, London SW17 0RE, UK; [b]Department of Physiology and Biophysics, Dalhousie University, Halifax, Nova Scotia, Canada

*Corresponding author: K. Török, Molecular and Clinical Sciences Research Institute, St George's, University of London, Cranmer Terrace, London SW17 0RE, UK
Phone: +44 2087255832; Fax: +44 2087253581; k.torok@sgul.ac.uk

Supplementary Materials

Supplementary Figure S1

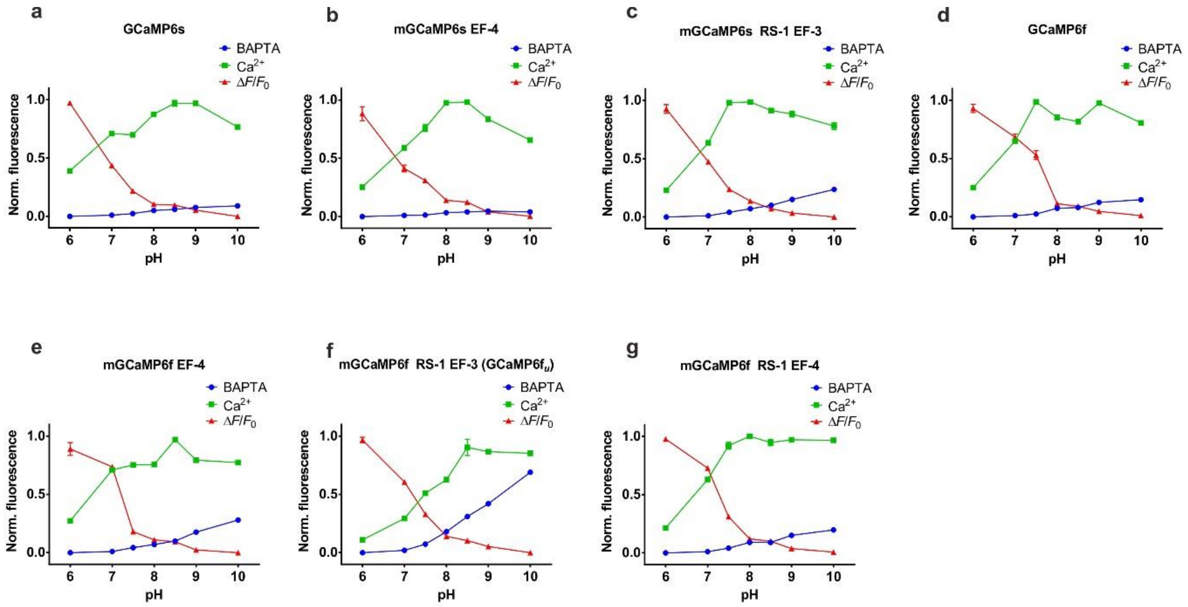

Supplementary Figure S1. pH sensitivity and pK_a determination of (**a**) GCaMP6s; (**b**) mGCaMP6s EF-4; (**c**) mGCaMP6s RS1 EF-3; (**d**) GCaMP6f; (**e**) mGCaMP6f EF-4; (**f**) mGCaMP6f RS1 EF-3 (GCaMP6f_u); (**g**) mGCaMP6f RS1 EF-4. Normalised fluorescence in presence of 1 mM Ca^{2+} (■) or 2 mM BAPTA (●); $\Delta F/F_0$ (▲).

Supplementary Figure S2

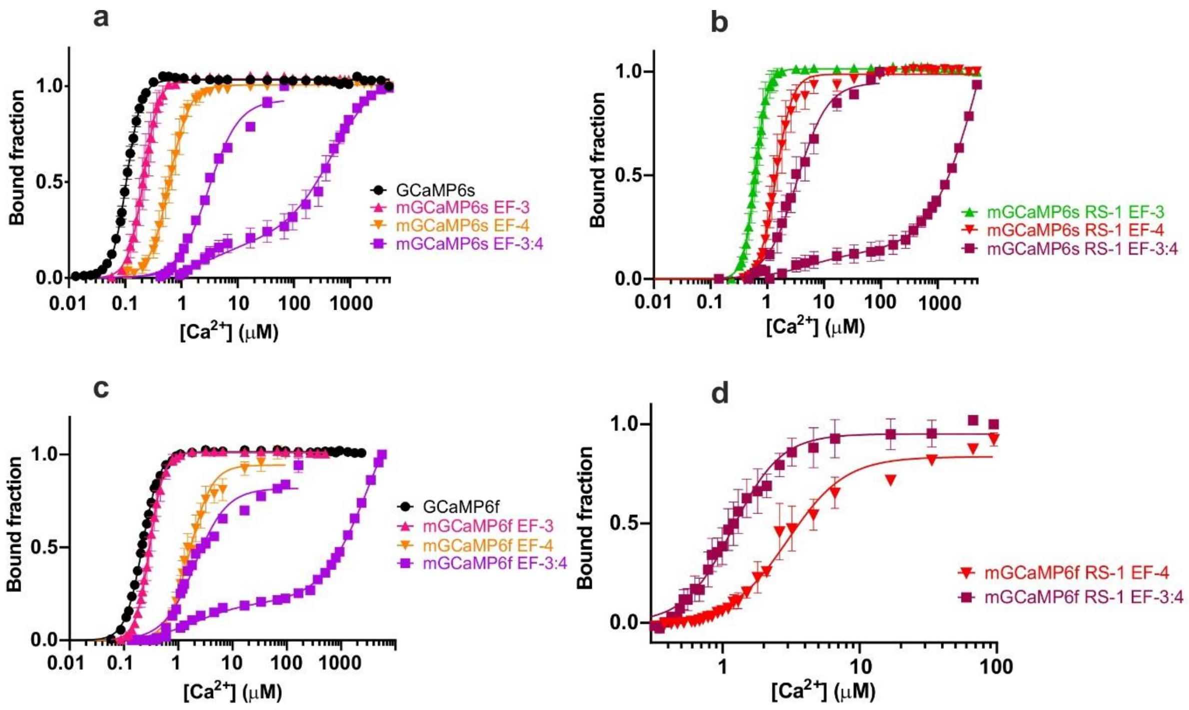

Supplementary Figure S2. Comparison of equilibrium Ca^{2+} binding of EF-hand and peptide mutant mGCaMP6s and mGCaMP6f to GCaMP6s and GCaMP6f (20 °C). Equilibrium Ca^{2+} titrations of (**a**) GCaMP6s (●), mGCaMP6s EF-3 (▲), mGCaMP6s EF-4 (▼) and mGCaMP6s EF-3:4 (■); (**b**) mGCaMP6s RS-1 EF-3 (▲), mGCaMP6s RS-1 EF-4 (▼) and mGCaMP6s RS-1 EF-3:4 (■); (**c**) GCaMP6f (●), mGCaMP6f EF-3 (▲), mGCaMP6f EF-4 (▼) and mGCaMP6f EF-3:4 (■); (**d**) mGCaMP6f RS-1 EF-4 (▼) and mGCaMP6f RS-1 EF-3:4 (■). Fluorescence changes are normalised to F_0 of 0 and F_{max} of 1 and fitted to the Hill equation. Fitted curves are represented by solid lines overlaying the data.

Supplementary Figure S3

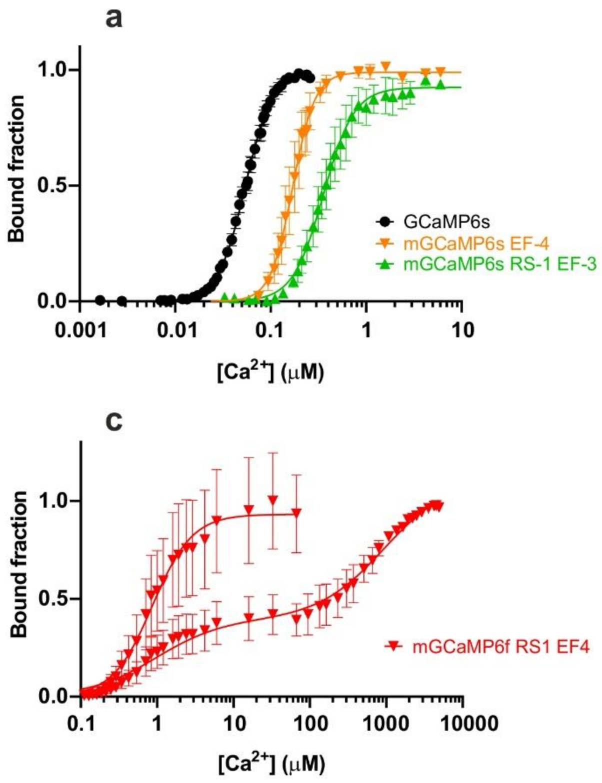

Supplementary Figure S3. Comparison of equilibrium Ca^{2+} binding of EF-hand and peptide mutant mGCaMP6s and mGCaMP6f to GCaMP6s and GCaMP6f (37 °C). Equilibrium Ca^{2+} titrations for (**a**) GCaMP6s (●), mGCaMP6s EF-4 (▼) and mGCaMP6s RS-1 EF-3 (▲); (**b**) GCaMP6f (●) and mGCaMP6f EF-4 (▼); (**c**) mGCaMP6f RS-1 EF-4 (▼). Fluorescence changes are normalized to F_0 of 0 and F_{max} of 1 and fitted to the Hill equation ((**a**) and (**b**)) and to a two-site binding model (**c**). Fitted curves are represented by solid lines overlaying the data points.

Supplementary Figure S4

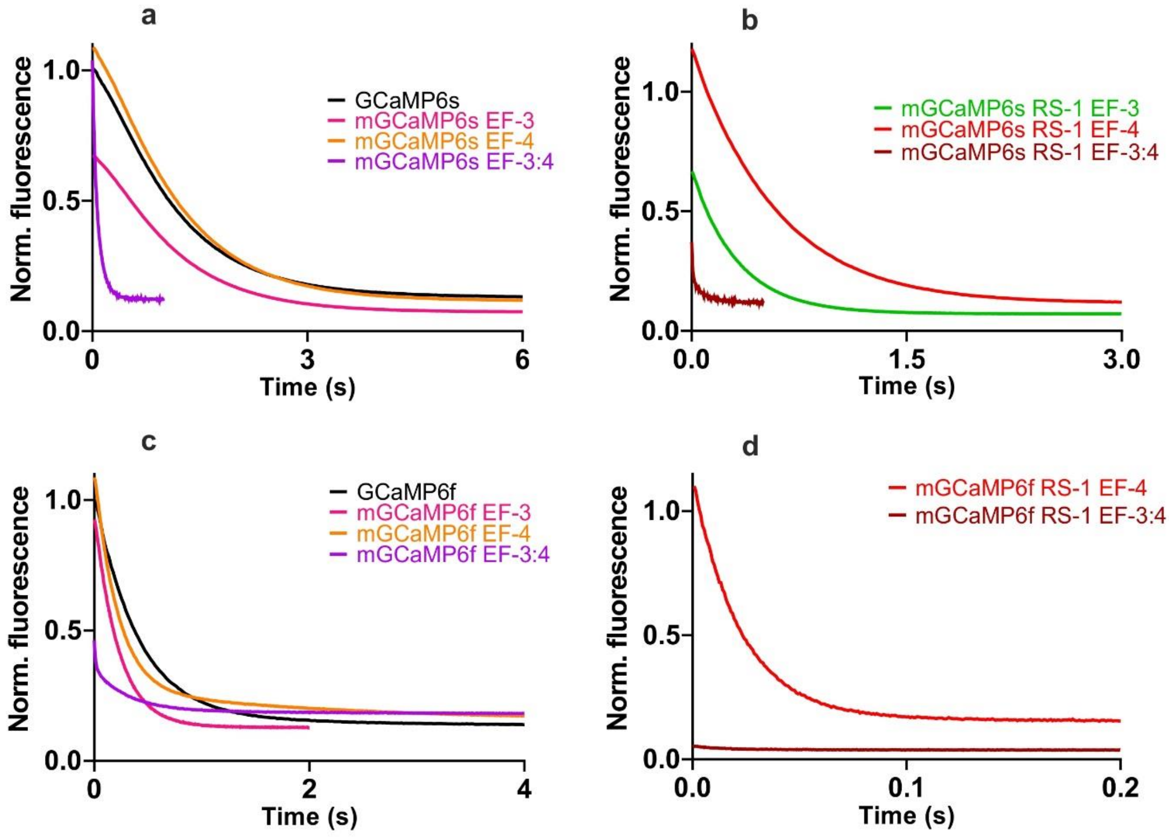

Supplementary Figure S4. Ca^{2+} dissociation kinetics of EF-hand and peptide mutant mGCaMP6s and mGCaMP6f compared with GCaMP6s and GCaMP6f (20 °C). Ca^{2+} dissociation time courses of (**a**) GCaMP6s (—), mGCaMP6s EF-3 (—), mGCaMP6s EF-4 (—) and mGCaMP6s EF-3:4 (—); (**b**) mGCaMP6s RS-1 EF-3 (—), mGCaMP6s RS-1 EF-4 (—) and mGCaMP6s RS-1 EF-3:4 (—); (**c**) GCaMP6f (—), mGCaMP6f EF-3 (—), mGCaMP6f EF-4 (—) and mGCaMP6f EF-3:4 (—); (**d**) mGCaMP6f RS-1 EF-4 (—) and mGCaMP6f RS-1 EF-3:4 (—). Fluorescence intensities are relative to Ca^{2+}-bound GCaMP6s (panels **a** and **b**) and to Ca^{2+}-bound GCaMP6f (panels **c** and **d**).

Supplementary Figure S5

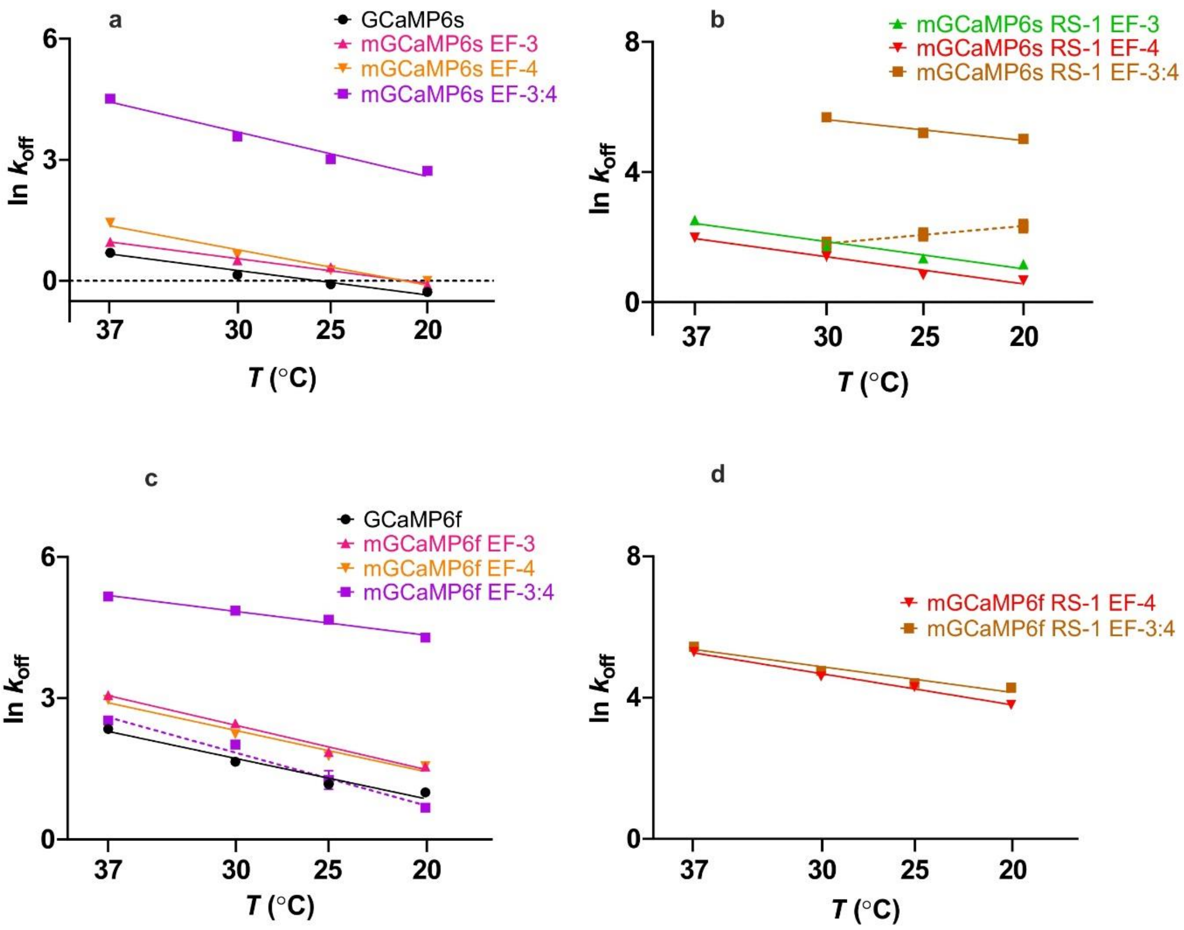

Supplementary Figure S5. Arrhenius plots of the observed rates for Ca^{2+} dissociation of EF-hand and peptide mutant mGCaMP6s and mGCaMP6f compared with GCaMP6s and GCaMP6f. (**a**) GCaMP6s (▬), mGCaMP6s EF-3 (▬), mGCaMP6s EF-4 (▬) and mGCaMP6s EF-3:4 (▬); (**b**) mGCaMP6s RS-1 EF-3 (▬), mGCaMP6s RS-1 EF-4 (▬) and mGCaMP6s RS-1 EF-3:4 (▬); (**c**) GCaMP6f (▬), mGCaMP6f EF-3 (▬), mGCaMP6f EF-4 (▬) and mGCaMP6f EF-3:4 (▬); (**d**) mGCaMP6f RS-1 EF-4 (▬) and mGCaMP6f RS-1 EF-3:4 (▬). For mGCaMP6 with biphasic kinetics, the slower rate is shown in dotted line and the faster rate is in solid line.

Supplementary Figure S6

	GCaMP6f	GCaMP6f RS-1 EF-3
Best-fit values		
Slope	-7322 ± 282.0	-5624 ± 202.9
Y-intercept when X=0.0	25.85 ± 0.9346	23.64 ± 0.6734
X-intercept when Y=0.0	0.003531	0.004204
1/slope	-0.0001366	-0.0001778
95% Confidence Intervals		
Slope	-7910 to -6734	-6045 to -5204
Y-intercept when X=0.0	23.90 to 27.80	22.25 to 25.04
X-intercept when Y=0.0	0.003514 to 0.003550	0.004142 to 0.004276
Goodness of Fit		
R square	0.9712	0.9722
Sy.x	0.0867	0.06573
Is slope significantly non-zero?		
F	674	768.7
DFn, DFd	1.000, 20.00	1.000, 22.00
P value	< 0.0001	< 0.0001
Deviation from zero?	Significant	Significant
Data		
Number of X values	7	7
Maximum number of Y replicates	4	4
Total number of values	22	24
Number of missing values	27	25
Equation	Y = -7322*X + 25.85	Y = -5624*X + 23.64

Supplementary Figure S6. Regression statistics for data in Fig. 1f.

Supplementary Figure S7

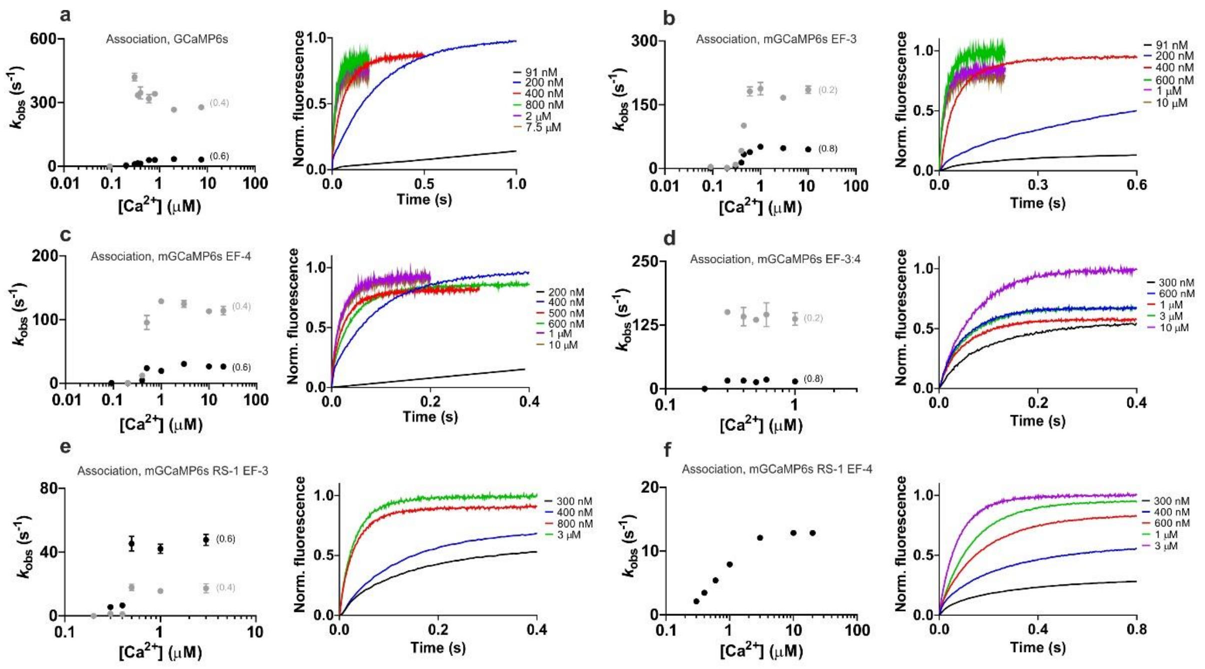

Supplementary Figure S7. Ca^{2+} association kinetics of GCaMP6s and its EF-hand and peptide mutants at 20 °C (**a**) GCaMP6s; (**b**) mGCaMP6s EF-3; (**c**) mGCaMP6s EF-4; (**d**) mGCaMP6s EF-3:4; (**e**) mGCaMP6s RS-1 EF-3; (**f**) mGCaMP6s RS-1 EF-4. In each panel, the left hand side plot shows the [Ca^{2+}] dependence of the observed rate(s) (k_{obs}) and on the right hand side stopped-flow records obtained at the specified final [Ca^{2+}] values are displayed. For mGCaMP6 with biphasic association kinetics (panels **a**-**e**), the dominant component (larger fluorescence amplitude) is shown in black. The relative amplitudes of the fast and slow phases are given in parentheses. Fluorescence changes are normalised to F_0 of 0 and maximum of 1.

Supplementary Figure S8

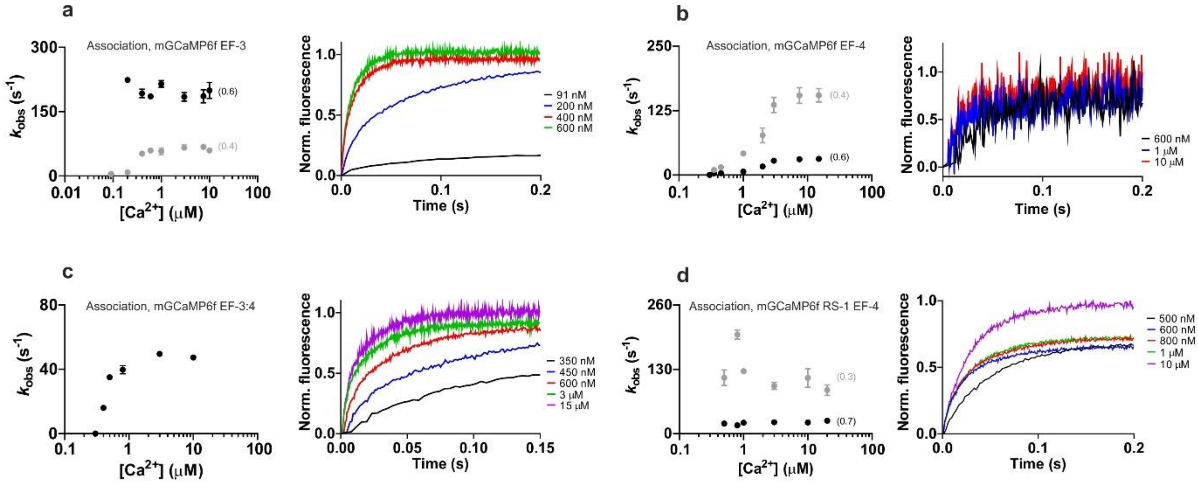

Supplementary Figure S8. Ca^{2+} association kinetics of the EF-hand and peptide mutants of GCaMP6f (20 °C). (**a**) mGCaMP6f EF-3; (**b**) mGCaMP6f EF-4; (**c**) mGCaMP6f EF-3:4; (**d**) mGCaMP6f RS-1 EF-4. In each panel, the left hand side plot shows the [Ca^{2+}] dependence of the observed rate(s) (k_{obs}) and on the right hand side stopped-flow records obtained at the specified final [Ca^{2+}] values are displayed. For mGCaMP6 with biphasic association kinetics (panels **a, b** and **d**), the dominant component (larger relative fluorescence amplitude) is shown in black. The relative amplitudes of the fast and slow phases are given in parentheses. Fluorescence changes are normalised to F_0 of 0 and maximum of 1.

Supplementary Figure S9

	GCaMP6f slow	GCaMP6f fast	GCaMP6f RS-1 EF-3
Best-fit values			
Slope	-2971 ± 300.0	-1602 ± 602.0	-7735 ± 1006
Y-intercept when X=0.0	13.72 ± 1.003	11.29 ± 2.046	31.33 ± 3.376
X-intercept when Y=0.0	0.004619	0.007049	0.00405
1/slope	-0.0003366	-0.0006243	-0.0001293
95% Confidence Intervals			
Slope	-3589 to -2353	-2914 to -290.2	-9879 to -5592
Y-intercept when X=0.0	11.66 to 15.79	6.833 to 15.75	24.14 to 38.52
X-intercept when Y=0.0	0.004399 to 0.004955	0.005405 to 0.02354	0.003899 to 0.004317
Goodness of Fit			
R square	0.7969	0.3711	0.7977
Sy.x	0.1221	0.1277	0.1737
Is slope significantly non-zero?			
F	98.09	7.082	59.14
DFn, DFd	1.000, 25.00	1.000, 12.00	1.000, 15.00
P value	< 0.0001	0.0207	< 0.0001
Deviation from zero?	Significant	Significant	Significant
Data			
Number of X values	8	5	5
Maximum number of Y replicates	5	4	4
Total number of values	27	14	17
Number of missing values	29	42	39
Equation	Y = -2971*X + 13.72	Y = -1602*X + 11.29	Y = -7735*X + 31.33

Supplementary Figure S9. Regression statistics for data in Fig. 2e.

Supplementary Figure S10

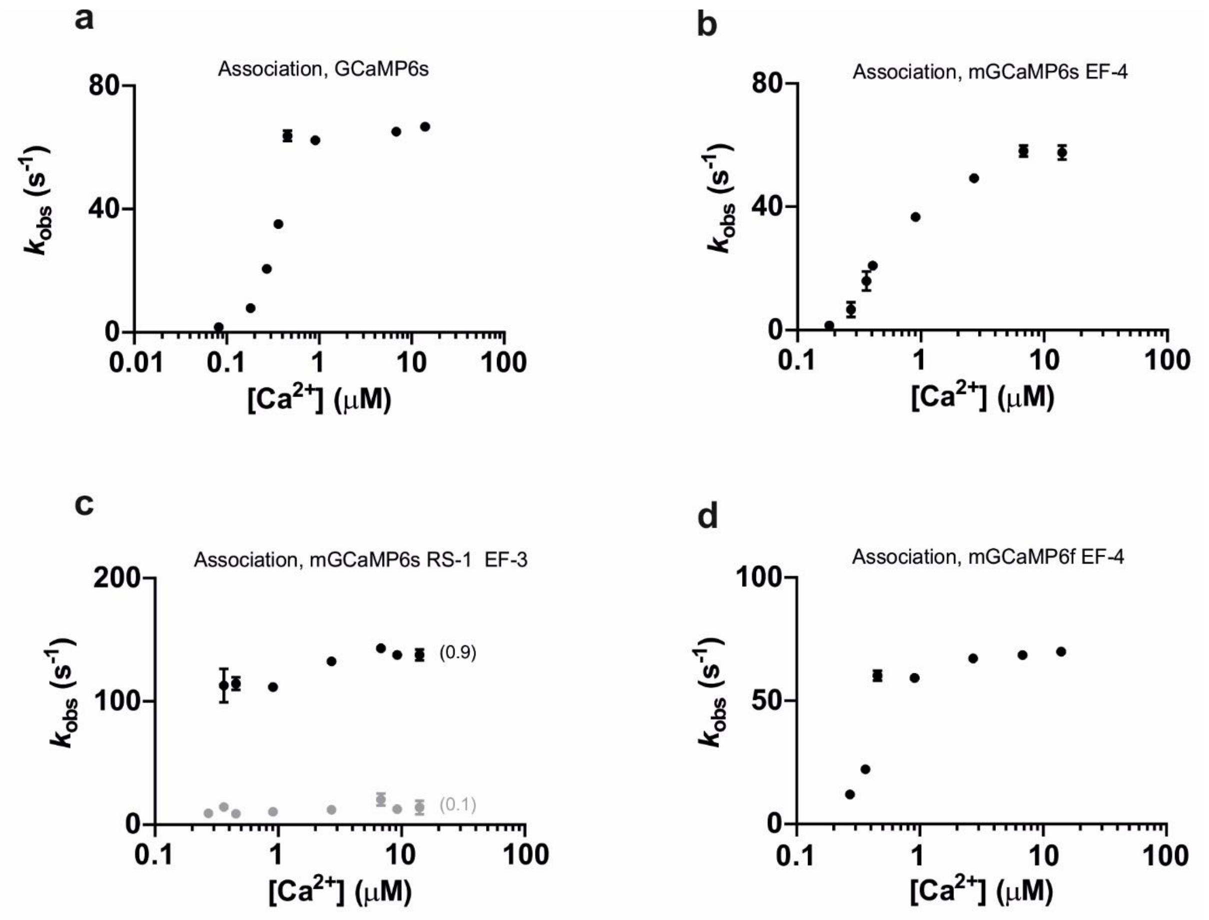

Supplementary Figure S10. Ca^{2+} dependence of association kinetics of selected probes at 37 °C. [Ca^{2+}] dependence of the observed rate(s) (k_{obs}) of (**a**) GCaMP6s; (**b**) mGCaMP6s EF-4; (**c**) mGCaMP6s RS-1 EF-3 and (**d**) mGCaMP6f EF-4. For mGCaMP6s RS-1 EF-3 biphasic association kinetics, the dominant component (larger relative fluorescence amplitude) is shown in black. The relative amplitudes of the fast and slow phases are given in parentheses.

Supplementary Figure S11

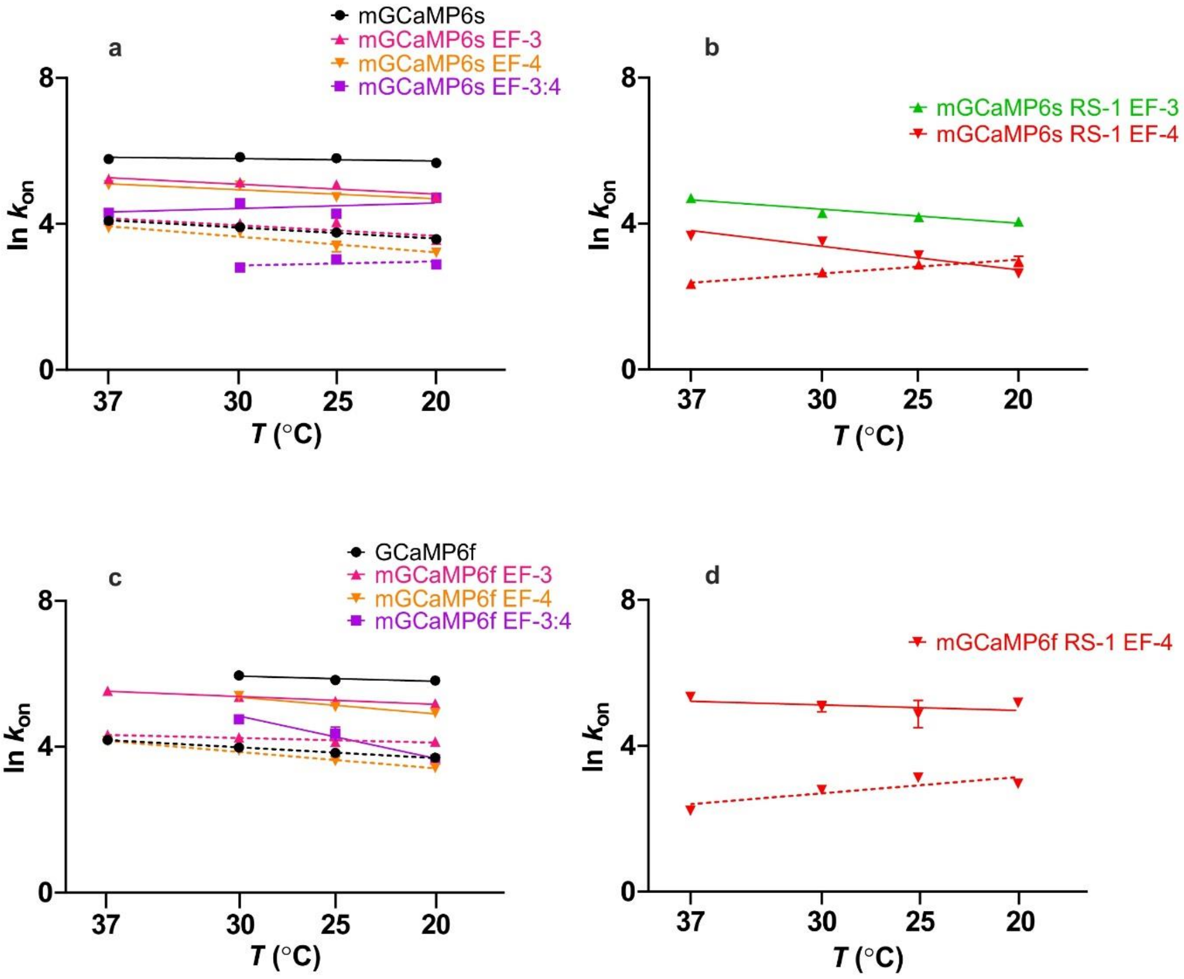

Supplementary Figure S11. Arrhenius plots of the observed rates for Ca^{2+} association of EF-hand and peptide mutant mGCaMP6s and mGCaMP6f compared with GCaMP6s and GCaMP6f. (**a**) GCaMP6s (▬), mGCaMP6s EF-3 (▬), mGCaMP6s EF-4 (▬) and mGCaMP6s EF-3:4 (▬); (**b**) mGCaMP6s RS-1 EF-3 (▬) and mGCaMP6s RS-1 EF-4 (▬); (**c**) GCaMP6f (▬), mGCaMP6f EF-3 (▬), mGCaMP6f EF-4 (▬) and mGCaMP6f EF-3:4 (▬); (**d**) mGCaMP6f RS-1 EF-4 (▬). For mGCaMP6 with biphasic kinetics, the slower rate is shown in dotted line and the faster rate is in solid line.

Supplementary Figure S12

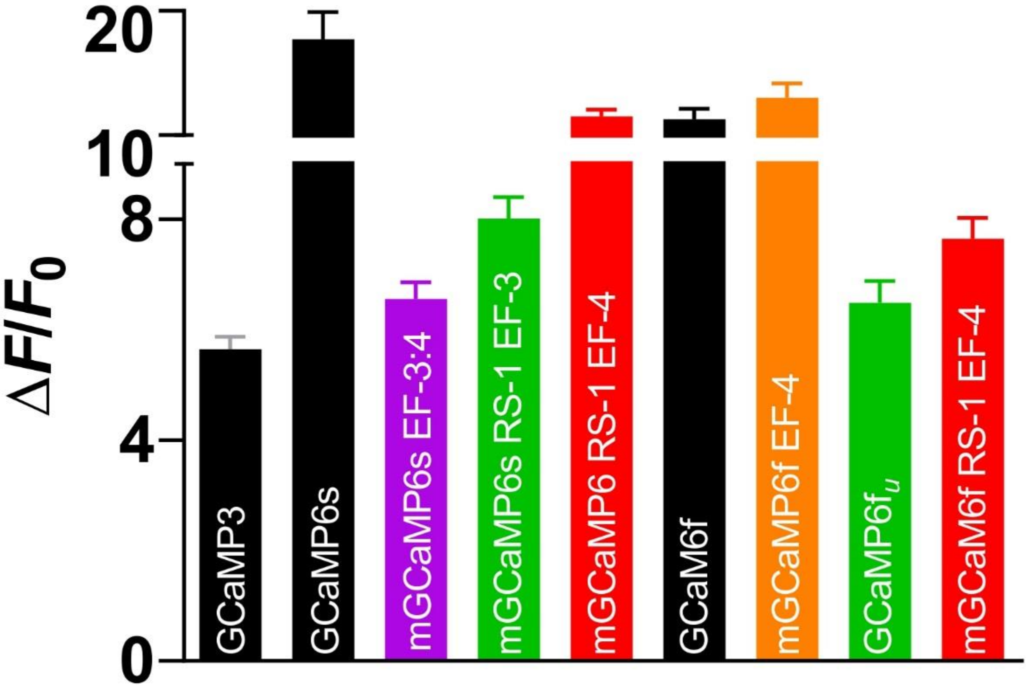

Supplementary Figure S12. Imaging of GCaMP6-derived probes in HEK293T cells. Fluorescence dynamic ranges of selected probes obtained following ionomycin stimulation.

Supplementary Figure S13

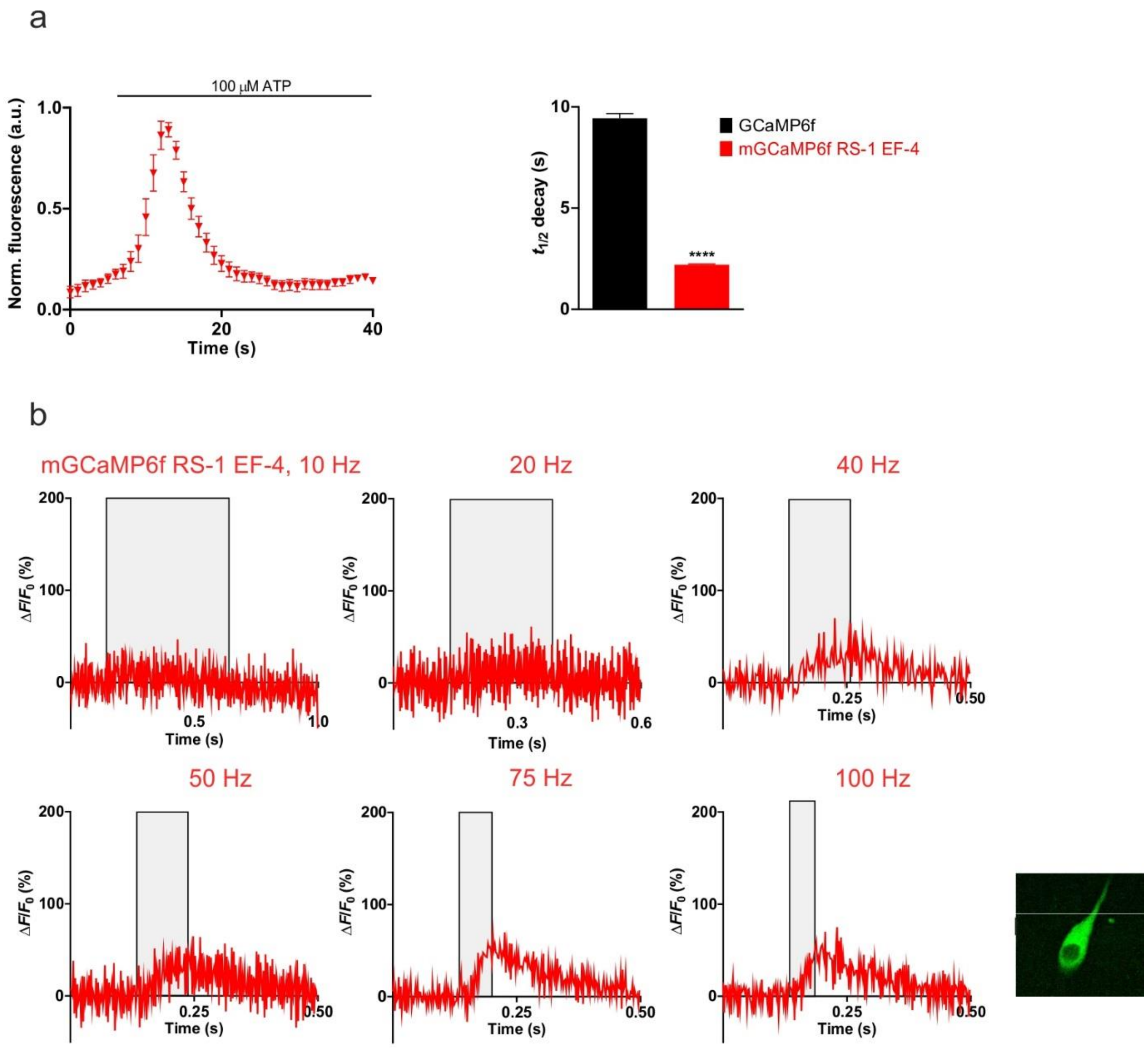

Supplementary Figure S13. Ca^{2+} response of mGCaMP6f RS-1 EF-4 in stimulated HEK293T cells and post-synaptic CA1 neurons. (**a**) Ca^{2+} transients were triggered by exposure of HEK293T cells to 100 μM ATP. Time course of response of mGCaMP6f RS-1 EF-4 was recorded with 1 s intervals. $t_{1/2}$ for mGCaMP6f RS-1 EF-4 of 2.2 ± 0.04 s was significantly different from that for GCaMP6f (9.4 ± 0.2 s). **(b)** Ca^{2+} response of mGCaMP6f RS-1 EF-4 in stimulated post-synaptic CA1 neurons is imaged by two-photon microscopy. GCaMP6f RS-1 EF-4 stimulated (3 cells, n number of recordings in brackets) by 5 AP-s at 10 Hz (n = 20), 20 Hz (n = 27), 40 Hz (n = 30), 50 Hz (n = 30), 75 Hz (n = 30) and 100 Hz (n = 30). Grey shaded areas indicate the duration of stimulation. The achieved maximum $\Delta F/F_0$ values are plotted against time. Inset image: representative image baseline expression of mGCaMP6f RS-1 EF-4 in CA1 pyramidal neurons with white line in the position of the line scan.

Supplementary Table S1. Summary of the biophysical characteristics of single EF-hand mutated mGCaMP6s and mGCaMP6f probes.

	$F_{r(-Ca^{2+})}$	$F_{r(+Ca^{2+})}$	$F_{r(+Ca^{2+})}$ / $F_{r(-Ca^{2+})}$	K_d (µM)	*n*	$k_{on(lim)}$[a] (s^{-1})	$t_{1/2(on)}$ (ms)	k_{off}[a] (s^{-1})	$t_{1/2(off)}$ (ms)
GCaMP6s **20 °C**	1.0 ± 0.1	27.3 ± 0.5	27.3 ± 0.9	0.11 ± 0.01	3.4 ± 0.1	306 ± 12 (0.4)	**2**	0.9	**769**
						31 ± 1 (0.6)	**22**		
GCaMP6s **37 °C**	1.0 ± 0.01	23.3 ± 0.1	23.3 ± 0.3	0.054 ± 0.005	3.0 ± 0.1	324 ± 23 (0.1)	**2**	2.0 ± 0.1	**346**
						59 ± 3 (0.9)	**12**		
mGCaMP6s EF-3 **20 °C**	1.1 ± 0.2	19.4 ± 0.2	18.1 ± 2.2	0.21 ± 0.01	3.2 ± 0.1	180 ± 5 (0.2)	**4**	0.9	**769**
						46 ± 2 (0.8)	**15**		
mGCaMP6s EF-3 **37 °C**	n.d.	n.d.	n.d.	n.d.	n.d.	187 ± 6 (0.5)	**4**	2.6 ± 0.1	**266**
						61 ± 3 (0.5)	**11**		
mGCaMP6s EF-4 **20 °C**	0.7 ± 0.1	20.0 ± 0.3	28.6 ± 1.1	0.61 ± 0.01	2.5 ± 0.1	120 ± 4 (0.4)	**6**	0.9	**769**
						27 ± 2 (0.6)	**26**		
mGCaMP6s EF-4 **37 °C**	0.9 ± 0.04	21.2 ± 1.0	23.9 ± 0.3	0.170 ± 0.005	3.7 ± 0.4	162 ± 22 (0.2)	**4**	4.2 ± 0.2	**165**
						49 ± 4 (0.8)	**14**		
mGCaMP6s RS-1 EF-3 **20 °C**	1.2 ± 0.1	21.1 ± 0.1	18.1 ± 0.7	0.62 ± 0.01	4.2 ± 0.1	45 ± 2 (0.6)	**15**	3.2 ± 0.1	**216**
						17 ± 1 (0.4)	**41**		
mGCaMP6s RS-1 EF-3 **37 °C**	1.0 ± 0.01	14.1 ± 0.1	14.1 ± 0.2	0.36 ± 0.01	2.7 ± 0.2	110 ± 1 (0.9)	**6**	13 ± 1	**53**
						11 ± 1 (0.1)	**63**		
mGCaMP6s RS-1 EF-4 **20 °C**	0.7 ± 0.1	21.8 ± 0.7	30.7 ± 1.1	1.4 ± 0.1	3.1 ± 0.1	13 ± 1	**53**	1.7	**407**
mGCaMP6s RS-1 EF-4 **37 °C**	n.d.	n.d.	n.d.	n.d.	n.d.	39 ± 3	**18**	39 ± 3	**18**
GCaMP6f **20 °C**	0.9 ± 0.1	14.4 ± 3.4	14.6 ± 2.4	0.22 ± 0.01	2.8 ± 0.1	315 ± 9 (0.3)	**2**	2.4 ± 0.1	**288**
						38 ± 1 (0.7)	**18**		
GCaMP6f **37 °C**	1.0 ± 0.1	13.8 ± 0.04	13.2 ± 0.1	0.088 ± 0.009	3.4 ± 0.1	66 ± 1	**10**	11 ± 1	**63**
mGCaMP6f EF-3 **20 °C**	1.3 ± 0.1	15.6 ± 0.3	12.4 ± 0.8	0.30 ± 0.03	3.4 ± 0.1	197 ± 6 (0.6)	**4**	4.5	**154**
						60 ± 2 (0.4)	**12**		
mGCaMP6f EF-3 **37 °C**	n.d.	n.d.	n.d.	n.d.	n.d.	255 ± 20 (0.6)	**3**	21 ± 1	**33**
						76 ± 3 (0.4)	**9**		
mGCaMP6f EF-4 **20 °C**	1.1 ± 0.1	21.2 ± 0.8	20.3 ± 2.0	1.6 ± 0.1	2.4 ± 0.1	155 ± 10 (0.4)	**4**	4.7	**147**
						31 ± 2 (0.6)	**22**		
mGCaMP6f EF-4 **37 °C**	1.0 ± 0.09	16.1 ± 0.3	15.8 ± 1	0.84 ± 0.03	1.8 ± 0.1	64 ± 1	**11**	20 ± 1	**35**
mGCaMP6f RS-1 EF-3 **20 °C** (GCaMP6f_u)	1.0 ± 0.1	5.1 ± 0.4	5.1 ± 0.1	0.89 ± 0.01	3.0 ± 0.1	142 ± 4	**4.9**	89 ± 1	**7.8**
mGCaMP6f RS-1 EF-3 **37 °C** (GCaMP6f_u)	0.9 ± 0.1	3.8 ± 0.2	4.1 ± 0.2	0.34 ±0.01	3.0 ± 0.3	546[b]	**1.3**	245 ± 10	**2.8**
mGCaMP6f RS-1 EF-4 **20 °C**	0.9 ± 0.1	16.9 ± 0.4	19.5 ± 0.4	3.0 ± 0.2 (0.8)[c]	2.1 ± 0.2	124 ± 9 (0.3)	**5.6**	44 ± 1	**16**
				229 ± 24 (0.2)[c]	1.2 ± 0.1	22 ± 1 (0.7)	**31**		
mGCaMP6f RS-1 EF-4 **37 °C**	0.9 ± 0.1	12.9 ± 0.1	14.6 ± 1.2	0.87 ± 0.14 (0.3)[c]	1.8 ± 0.4	210 ± 10 (0.7)	**3.3**	199 ± 3	**3.5**
				961 ± 212 (0.7)[c]	1	9 ± 1 (0.3)	**77**		

[a]Biphasic association kinetic records were fitted with two exponentials. The rate of each phase is given with the relative amplitudes in parentheses. [b]Measurements were made at 20, 25 and 30°C. The rate was too fast to measure at 37°C, thus value was extrapolated from the Arrhenius plot assuming the gradient remaining unchanged (**Fig. 1f**). [c]Values in parentheses represent relative fluorescence amplitudes of two binding sites. n.d. denotes not determined.

Supplementary Table S2. Summary of the biophysical characteristics of double EF-hand mutated mGCaMP6s and mGCaMP6f probes.

	$F_{r(-Ca^{2+})}$	$F_{r(+Ca^{2+})}$	$F_{r(+Ca^{2+})}$ / $F_{r(-Ca^{2+})}$	K_d (μM)	n	$k_{on(lim)}$ (s^{-1})	$t_{1/2(on)}$ (ms)	k_{off} (s^{-1})	$t_{1/2(off)}$ (ms)
mGCaMP6s EF-3:4 **20 °C**	1.0 ± 0.1	5.8 ± 2.3	5.8 ± 1.2	2.7 ± 0.1 (0.2)	1.8 ± 0.1	141 ± 8 (0.2)[a]	**5**	13.4 ± 0.1	**52**
		19.0 ± 0.2	19.1 ± 0.3	466 ± 35 (0.8)	1	16 ± 1 (0.8)[a]	**43**		
mGCaMP6s EF-3:4 **37 °C**	n.d.	n.d.	n.d.	n.d.	n.d.	74 ± 2	**9**	93 ± 9	**7**
mGCaMP6s RS-1 EF-3:4 **20 °C**	1.1 ± 0.1	1.3 ± 0.4	1.3 ± 0.3	3.3 ± 0.2 (0.2)[c]	1.8 ± 0.1	n.d.	n.d.	135 ± 3 (0.6)[a]	**5**
		5.8 ± 0.3	5.4 ± 0.4	6335 ± 874 (0.8)[c]	1			12 ± 1 (0.4)[a]	**58**
mGCaMP6s RS-1 EF-3:4 **37 °C**	n.d.	n.d.	n.d.	n.d.	n.d.	n.d.	n.d.	420[b]	**2**
								4.2*	**165**
mGCaMP6f EF-3:4 **20 °C**	0.9 ± 0.1	2.0 ± 0.1	2.2 ± 0.2	2.2 ± 0.1 (0.3)[c]	1.7 ± 0.1	48 ± 1	**14**	67 ± 5 (0.4)[a]	**10**
		5.7 ± 0.3	6.2 ± 0.4	3606 ± 261 (0.7)[c]	1			3.0 ± 0.1 (0.6)[a]	**231**
mGCaMP6f EF-3:4 **37 °C**	n.d.	n.d.	n.d.	n.d.	n.d.	276.0[b]	**3**	174 ± 5 (0.8)[a]	**4**
								13 ± 1 (0.2)[a]	**53**
mGCaMP6f RS-1 EF-3:4 **20 °C**	1.0 ± 0.1	1.4 ± 0.1	1.4 ± 0.1	1.2 ± 0.1	2.5 ± 0.2	n.d.	**n.d**	65 ± 3	**11**
mGCaMP6f RS-1 EF-3:4 **37 °C**	n.d.	n.d.	n.d.	n.d.	n.d.	n.d.	n.d.	233 ± 18	**3**

[a]Biphasic kinetic records were fitted with two exponentials. The rate of each phase is given with the relative amplitudes in parentheses. [b]Measurements were made at 20, 25 and 30°C. The rates was too fast to measure at 37°C, thus the value was extrapolated from the Arrhenius plot assuming the gradient remaining unchanged (**Supplementary Fig. S4b,S9c**). [c]Values in parentheses represent relative fluorescence amplitudes of two binding sites. Error represents the standard error of the estimate for the average of three records. n.d. denotes not determined.

Supplementary Table S3

	k_{+1} (μM^{-1} s^{-1})	k_{-1} (s^{-1})	k_{+2} (μM^{-1} s^{-1})	k_{-2} (s^{-1})	k_{+3} (μM^{-1} s^{-1})	k_{-3} (s^{-1})	k_{+4} (s^{-1})	k_{-4} (s^{-1})	k_{+5} (μM^{-1} s^{-1})	k_{-5} (s^{-1})	k_{+6} (μM^{-1} s^{-1})	k_{-6} (s^{-1})	k_{+7} (μM^{-1} s^{-1})	k_{-7} (s^{-1})	k_{+8} (s^{-1})	k_{-8} (s^{-1})	F_1	F_2	K_d (μM)
GCaMP6f 20 °C	770	1100	15038	935	786	1.0E-02	395	1210	84	252	3464	980	2185	19	548	5.0E-03	54	14	0.22
GCaMP6f 37 °C	770	1100	15038	935	786	2.1E-05	408	1242	84	252	3464	264	2185	19	548	3.8E-05	59	14	0.088
mGCaMP6f RS-1 EF-3 (GCaMP6f_u) 20 °C	770	1100	15038	935	337867	2.9E-03	138	37	-	-	84	23651	963	1793	1232	8.0E-04	20	15	0.89
mGCaMP6f RS-1 EF-3 (GCaMP6f_u) 30 °C	770	1100	15038	935	337867	2.9E-03	223	71	-	-	84	23651	963	1793	1232	1.8E-03	16	15	0.57
